# Dissecting the epigenetic regulation of the fetal hemoglobin genes to unravel a novel therapeutic approach for β-hemoglobinopathies

**DOI:** 10.1093/nar/gkaf637

**Published:** 2025-07-10

**Authors:** Simone Amistadi, Letizia Fontana, Chiara Magnoni, Tristan Felix, Matteo Kane Charvin, Pierre Martinucci, Candice Gautier, Lilian Greau, Bettina Bessières, Panagiotis Antoniou, Oriana Romano, Eric Allemand, Claudio Mussolino, Annarita Miccio

**Affiliations:** Laboratory of Chromatin and Gene Regulation during Development, Imagine Institute, INSERM UMR1163, Paris Cité University, Paris, 75015, France; Laboratory of Chromatin and Gene Regulation during Development, Imagine Institute, INSERM UMR1163, Paris Cité University, Paris, 75015, France; Laboratory of Chromatin and Gene Regulation during Development, Imagine Institute, INSERM UMR1163, Paris Cité University, Paris, 75015, France; Laboratory of Chromatin and Gene Regulation during Development, Imagine Institute, INSERM UMR1163, Paris Cité University, Paris, 75015, France; Laboratory of Chromatin and Gene Regulation during Development, Imagine Institute, INSERM UMR1163, Paris Cité University, Paris, 75015, France; Laboratory of Chromatin and Gene Regulation during Development, Imagine Institute, INSERM UMR1163, Paris Cité University, Paris, 75015, France; Laboratory of Cellular and Molecular Mechanisms of Hematological Disorders and Therapeutic Implications, Imagine Institute, INSERM UMR1163, Paris, 75015, France; Genomics Core Facility, Institut Imagine-Structure Fédérative de Recherche Necker, INSERM U1163 et INSERM US24/CNRS UAR3633, Paris Cité University, Paris, 75015, France; Service de Médecine Génomique des Maladies Rares, Hôpital Necker-Enfants Malades, Paris, 75015, France; Laboratory of Chromatin and Gene Regulation during Development, Imagine Institute, INSERM UMR1163, Paris Cité University, Paris, 75015, France; Department of Molecular Medicine, University of Padova, Padova, 35122, Italy; Laboratory of Cellular and Molecular Mechanisms of Hematological Disorders and Therapeutic Implications, Imagine Institute, INSERM UMR1163, Paris, 75015, France; Institute for Transfusion Medicine and Gene Therapy, Medical Center – University of Freiburg, 79098, Freiburg, Germany; Laboratory of Chromatin and Gene Regulation during Development, Imagine Institute, INSERM UMR1163, Paris Cité University, Paris, 75015, France

## Abstract

Beta-hemoglobinopathies are severe genetic diseases caused by mutations affecting the production of the adult β-globin chain. The clinical severity is mitigated by the co-inheritance of mutations that reactivate the production of the fetal β-like γ-globin in adults. However, the epigenetic mechanisms underlying the adult-to-fetal hemoglobin (HbA-to-HbF) switching are still not fully understood. Here, we used epigenome editing technologies to dissect the molecular mechanisms underlying γ- and β-globin gene regulation and to develop novel potential therapeutics for β-hemoglobinopathies. Targeted removal of DNA methylation by dCas9-Tet1 (alone or together with the deposition of histone acetylation by CBP-dCas9) at the fetal promoters led to efficient and durable γ-globin reactivation, demonstrating that DNA methylation is a driver for HbF repression. This strategy, characterized by high specificity and a good safety profile, led to a substantial correction of the pathological phenotype in erythroid cells from patients with sickle cell disease.

## Graphical Abstract

**Graphical Abstract:**
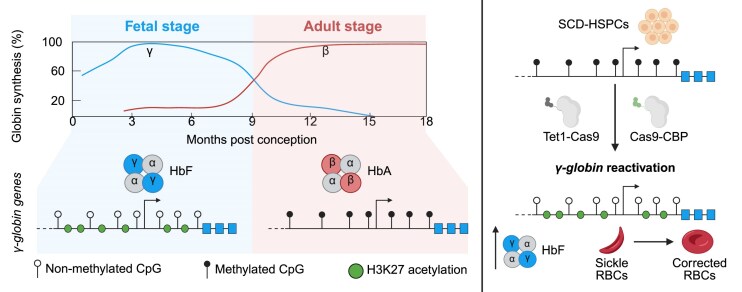

## Introduction

Sickle cell disease (SCD) is a genetic disorder caused by a point mutation in the *HBB* gene leading to the formation of the sickle hemoglobin (HbS), which polymerizes under hypoxic conditions. This leads to red blood cell (RBC) sickling, anemia, and multi-organ damage [1, 2]. Beta-thalassemia is caused by >300 mutations reducing the expression of the *HBB* gene and leading to anemia [3–5]. The severity of β-hemoglobinopathies is dampened by a benign genetic condition named hereditary persistence of fetal hemoglobin (HPFH) [6]. HPFH mutations in the identical *HBG1* and *HBG2* γ-globin promoters either generate *de novo* DNA motifs recognized by transcriptional activators (e.g. KLF1) [7–9] or disrupt binding sites for transcriptional repressors (e.g. LRF and BCL11A) [10]. This results in the reactivation of the γ-globin gene and in the formation of fetal hemoglobin (HbF) in adulthood.

Besides transcription factors, chromatin-remodeling and modifier complexes, such as the Nucleosome Remodeling and Deacetylation repressor complex (NuRD), play a critical role in the γ-to-β-globin switch [11–13]. These complexes include enzymes capable of methylating or demethylating DNA. DNA methyltransferases (DNMT) transfer methyl groups onto the fifth carbon atom of the cytosine of a CpG site. DNMT3A and DNMT3B mediate the *de novo* deposition of methyl groups in a non-methylated 5′-CpG-3′ and symmetrically to the non-methylated 3′-GpC-5′ of the complementary strand [14]. DNMT1 is responsible for the maintenance of the methylation of asymmetrically semi-methylated CpG sites upon DNA replication [15]. The ten-eleven translocation methyl-cytosine dioxygenase (TET) proteins (TET1, TET2, and TET3) are responsible for DNA demethylation [16, 17]. In particular, they mediate the removal of the methyl group from a 5-methyl-cytosine (5-mC) through the generation of a 5-hydroxy-methyl-cytosine (5-hmC) intermediate [16, 17]. High 5-mC levels normally characterize transcriptionally inactive promoters, while hypomethylated promoters and those bearing the 5-hmC intermediate are generally highly expressed [18].

Within the γ-globin promoters, 7 CpG sites (−252, −162, −53, −50, +6, +17, +50; position from the transcription start site, TSS) are differentially methylated in adult bone marrow (BM) erythroid cells compared to cord blood and fetal liver erythrocytes [19–21]. In line with the hypothesis that promoter CpG methylation is associated with gene silencing, erythroid cells obtained from adult human hematopoietic stem and progenitor cells (HSPCs) reveal highly methylated CpGs in the *HBG* promoters. In contrast, fetal cells show a general hypomethylation of the same CpGs [19] similar to those from β-thalassemia patients (that express mainly HbF) [21] or individuals with HPFH mutations [22]. However, whether DNA methylation directly influences *HBG* expression, or it is a mere consequence of the recruitment of transcription factors and chromatin-remodeling and modifier complexes interacting with DNMT (e.g. NuRD [11, 13]) is still debated [23]. Interestingly, a missense mutation in *DNMT1* was associated with HbF reactivation in erythroid cells differentiated from adult HSPCs. This could be potentially due to the diminished recruitment of DNMT1 at the γ-globin promoters and the consequent reduced CpG methylation levels [24]. Furthermore, knockdown of the Methyl-CpG Binding Domain Protein 2 (a member of the NuRD repressor complex), which occupies the methylated *HBG* promoters in adult erythroid cells, leads to HbF reactivation [25, 26], suggesting that CpG methylation at the *HBG* promoters favors NuRD recruitment and gene repression.

In addition to DNA methylation, histone modifiers, such as histone acetyltransferases (HATs) and histone deacetylases (HDACs), are implicated in the hemoglobin switching. In human primary cells, when a β-like globin gene is activated (e.g. the γ-globin gene in fetal erythroblasts), histone residues (H3 lysine 9, H3K9 and H3 lysine 27, H3K27) in the promoter regions are highly acetylated, contributing to the generation of an open chromatin state associated with active transcription [19, 27–29].

Drugs such as the DNMT inhibitor decitabine or the HDAC inhibitor vorinostat have been reported to increase γ-globin expression [30, 31]. However, the mode of action of these drugs remains uncertain, and a causal relationship between the induced epigenetic changes and γ-globin reactivation has not been demonstrated. Moreover, their non specific, global effects pose serious safety concerns for clinical use for patients with β-hemoglobinopathies. Similarly, downregulation of genes encoding components of chromatin-remodeling and modifier complexes is associated with HbF upregulation, but with few exceptions, such as CHD4 downregulation, this leads to impaired cell fitness, as these factors play a pleiotropic role in the establishment and maintenance of the cellular epigenetic and transcriptional profiles [25, 32].

Gene therapy approaches based on the transplantation of autologous, genetically modified HSPCs have been investigated as a treatment option for patients lacking an allogeneic compatible donor [33]. CRISPR–Cas9 nuclease and base editor strategies have been developed to introduce HPFH and HPFH-like mutations in the *HBG1*/*2* promoters [34–40]. These technologies rely on the cleavage of one or both DNA strands, a process particularly critical in HSPCs, which are notoriously highly sensitive to DNA damage. This sensitivity becomes even more pronounced in cases involving multiple on-target events, such as targeting *HBG*, which has two copies on chromosome 11, or when both on-target and off-target events occur simultaneously [41–43]. In this context, CRISPR–Cas9 editing of human HSPCs induces a DNA damage response that can lead to apoptosis [44, 45] via p53-dependent cell toxicity and cell cycle arrest [46]. Furthermore, the generation of double-strand breaks (DSBs) is associated with genotoxicity, such as the occurrence of genomic deletions, inversions, translocations, chromosome loss, and chromothripsis [47–50].

Recently, epigenome editors have been developed and used for precise gene modulation [51–53]. These tools are composed of a DNA binding domain (e.g. a catalytically inactive dead Cas9, dCas9) fused with effectors that are able to introduce epigenetic modifications such as histone acetylation (e.g. dCas9-P300, dCas9-CBP) or DNA demethylation (e.g. dCas9-Tet1) [54, 55]. In the context of β-hemoglobinopathies, epigenome editors containing HATs have been used to reactivate γ-globin expression in HEK293T cells [54, 56–58], while targeted modulation of DNA methylation has never been tested as a potential therapeutic tool for *HBG* induction. Furthermore, efficacy and safety of epigenome editors have never been assessed in primary HSPCs neither *in vitro* nor *in vivo*.

In this study, we characterized the epigenetic regulation of the *HBG* promoters by assessing the role of DNA methylation and histone acetylation in modulating γ-globin expression. Furthermore, we demonstrated that epigenome editing represents a novel, efficacious and safe strategy to treat primary HSPCs from β-hemoglobinopathy patients and achieve HbF reactivation in their erythroid progeny.

## Materials and methods

### Plasmids

Plasmids used in this study include dCas9 (Addgene, #100091), Tet1-dCas9 (Addgene, #136650), and dCas9-CBPcore (Addgene, #179543). The plasmids used as templates for messenger RNA (mRNA) *in vitro* transcription (ivt) were obtained by substituting the coding sequence of pCMV-T7-ABE8e-nSpRY-P2A-EGFP (Addgene, #185912) with the desired editor through Gibson assembly cloning (NEB) following manufacturer’s recommendation. All the single guide RNAs (sgRNAs) were cloned into the pMA128 (provided by M. Amendola, Genethon, France).

### sgRNA design

To generate the plasmids expressing the sgRNAs recognizing the specific protospacer (Table 1), oligonucleotides were annealed, and the duplexes were ligated into the BbsI-digested MA128 plasmid. For RNA-mediated base editing, we used chemically modified synthetic sgRNAs harboring 2′-*O*-methyl analogs and 3′-phosphorothioate nonhydrolyzable linkages at the first three 5′ and 3′ nucleotides (Synthego).

**Table 1.** List of sgRNA spacer sequences

sgRNA	spacer sequence (5′ to 3′)	PAM
−211	TTAAGCAGCAGTATCCTCTT	GGG
−197	ATTGAGATAGTGTGGGGAAG	GGG
−165	ATGCAAATATCTGTCTGAAA	CGG
−115	CTTGTCAAGGCTATTGGTCA	AGG
−28	GGCTAGGGATGAAGAATAAA	AGG

### mRNA *in vitro* transcription

Ten micrograms of editor-expressing plasmids were digested overnight with 20 units of a restriction enzyme that cleaves once after the stop codon. The linearized plasmids were purified using the PCR purification kit (QIAGEN) and were eluted in 30 μl of DNase/RNase-free water. One microgram of linearized plasmid was used as template for the ivt reaction (MEGAscript, Ambion). The ivt protocol was modified as follows. The GTP nucleotide solution was used at a final concentration of 3.0 mM instead of 7.5 mM and the anti-reverse cap analog *N*^7^-methyl-3′-*O*-methyl-guanosine-5′-triphosphate-5′-guanosine (ARCA, Trilink) was used at a final concentration of 12.0 mM resulting in a final ratio of Cap:GTP of 4:1 that allows efficient capping of the mRNA. The incubation time for the ivt reaction was reduced to 30 min. The polyadenylation step was performed following manufacturer’s guidelines (PolyA tailing kit, Ambion). mRNA was precipitated using lithium chloride and resuspended in TE buffer in a final volume that allowed us to achieve a concentration of >1 μg/μl. The mRNA quality was evaluated using Tapestation 2200 (Agilent).

### Cell line culture and transfection

HEK293T cells were cultured in Dulbecco’s modified Eagle’s medium supplemented with 10% of fetal bovine serum (Lonza) and 1% penicillin/streptomycin (Gibco). For transfections, 1.5 × 10^5^ cells/condition were seeded 24 h before in a 24-well plate and then transfected with polyethylenimine (PEI) with epigenome editor-expressing plasmids and sgRNA-containing plasmid in a ratio of 3:1 with a total of 1 ug DNA transfected. In the combinations with less sgRNAs, the MA128 plasmid was used to reach 1 ug of total DNA per transfection. Twenty-four hours after transfection the medium was changed. Cells were collected 72 h after transfection (or at indicated time points for time course experiment) for reverse transcription quantitative polymerase chain reaction (RT-qPCR) analysis.

### HSPC purification and culture

Adult human non-mobilized peripheral blood or BM CD34^+^ HSPCs from SCD patients, adult human mobilized peripheral blood CD34^+^ HSPCs from healthy donors, and fetal human CD34^+^ HSPCs from fetal liver of healthy donors were obtained from the “Hôpital Necker-Enfants malades” Hospital (Paris, France). BM CD34^+^ HSPCs derive from young SCD patients (3 to 12 years old) that underwent hematopoietic stem-cell transplantation. Before the transplantation, they had similar clinical features with recurrent acute chest syndrome and vaso-occlusive crisis. One out of three was under hydroxyurea treatment. Written informed consent was obtained from all adult subjects. All experiments were performed in accordance with the Declaration of Helsinki. The study was approved by the regional investigational review board (reference: DC-2024-6899, CPP Ile-de-France II “Hôpital Necker-Enfants malades”). HSPCs were purified by immunomagnetic selection with MACS columns (Miltenyi Biotec) after immunostaining with the CD34 MicroBead Kit (Miltenyi Biotec). Forty-eight hours before transfection, CD34^+^ cells were thawed and cultured at a concentration of 5 × 10^5^ cells/ml in StemSpan (STEMCELL Technologies) supplemented with penicillin/streptomycin (Gibco), 250 nM StemRegenin (STEMCELL Technologies), 38 nM UM171 (STEMCELL Technologies), and the following recombinant human cytokines (PeproTech): human stem cell factor (SCF) (300 ng/ml), Flt-3L (300 ng/ml), thrombopoietin (TPO) (100 ng/ml), and interleukin-3 (IL-3) (60 ng/ml).

### RNA transfection

0.5 × 10^5^ to 2 × 10^5^ CD34^+^ HSPCs per condition were transfected with 2 μg of each enzyme-encoding mRNA and a mix of synthetic sgRNAs (2 μg each). The P3 Primary Cell 4D-Nucleofector X Kit S (Lonza) was used with the CA137 program (Nucleofector 4D). Cells transfected with TE buffer or with the enzyme-encoding mRNA only served as negative controls.

### Colony-forming cell assay

CD34^+^ HSPCs were plated at a concentration of 1000 cells/m in a methylcellulose-based medium (GFH4435, Stem Cell Technologies) under conditions supporting erythroid (BFU-E) and granulocyte/monocyte (CFU-GM) differentiation. BFU-E and CFU-GM colonies were counted after 14 days. Colonies were randomly picked and collected as bulk populations (containing at least 25 colonies) to evaluate globin expression by RT-qPCR and hemoglobin production by HPLC.

### HSPC differentiation

Transfected HSPCs were differentiated into mature RBCs using a three-phase erythroid differentiation protocol. During the first phase (day 0 to day 6), cells were cultured in a basal erythroid DOUAY medium [59] supplemented with 100 ng/ml recombinant human SCF (PeproTech), 5 ng/ml recombinant human IL-3 (PeproTech), 3 IU/ml EPO Eprex (Janssen-Cilag), and 10^−6^ M hydrocortisone (Sigma). During the second phase (day 6 to day 9), cells were co-cultured with MS-5 stromal cells [59] in the basal erythroid DOUAY medium supplemented with 3 IU/ml EPO Eprex (Janssen-Cilag). During the third phase (day 9 to day 13), cells were co-cultured with stromal MS-5 cells in a basal erythroid DOUAY medium without cytokines. During the last phase (day 13 to day 20), heat-inactivated human AB serum (10%) was added to the basal erythroid DOUAY medium. Erythroid differentiation was monitored by flow cytometry analysis of CD36, CD71, CD235a, BAND3, and CD49d erythroid surface markers and analysis of enucleated cells using the DRAQ5 double-stranded DNA dye. 7AAD was used to identify live cells.

### Flow cytometry analysis

HSPC-derived erythroid cells were fixed with 0.05% cold glutaraldehyde and permeabilized with 0.1% Triton X-100. After fixation and permeabilization, cells were stained with an antibody recognizing CD235a erythroid surface marker (1/100 PE-Cy7-conjugated anti-CD235a antibody, 563666, BD Pharmingen) and an antibody recognizing HbF (1/5 FITC-conjugated anti-HbF antibody, clone 2D12 552829 BD). Flow cytometry analysis of CD36, CD71, CD235a, BAND3, and CD49d erythroid surface markers was performed using a V450-conjugated anti-CD36 antibody (1/20 561535, BD Horizon), a FITC-conjugated anti-CD71 antibody (1/50 555536, BD Pharmingen), a PE-Cy7-conjugated anti-CD235a antibody (1/100 563666, BD Pharmingen), a PE-conjugated anti-BAND3 antibody (1/50 9439, IBGRL), and an APC-conjugated anti-CD49d antibody (1/20 559881, BD). Flow cytometry analysis of enucleated or viable cells was performed using double-stranded DNA dyes DRAQ5 (65-0880-96, Invitrogen) and 7AAD (559925, BD), respectively. All analyses were performed using the Novocyte Flow Cytometry system (Agilent). Data were analyzed using the FlowJo (BD Biosciences) software.

### Western blot

HSPCs, HSPC-derived erythroblasts, and K562 cells were harvested after electroporation at indicated time points (12 h, day 1, day 2, day 6). Cells were lysed with RIPA buffer (Thermo Fisher Scientific) supplemented with cOmplete^™^ Mini EDTA-free Protease Inhibitor Cocktail (Roche) and incubated on ice for 30 min. Lysates were sonicated using the Sonics Vibra-Cell VCX750 (2 cycles of 10 s, 9 s ON and 1 s OFF, amplitude 50%). Following sonication, samples were centrifuged at 13 000 × *g* for 12 min at 4°C and the soluble fractions were collected. Proteins were quantified with Pierce BCA Protein Assay Kit (Thermo Fisher Scientific) following manufacturer’s instructions. At least 30 μg of total protein were mixed with sodium dodecyl sulfate (SDS) loading buffer, heated, and loaded on NuPAGE™ Bis-Tris Mini Protein Gels, 4%–12% (Thermo Fisher Scientific) using NuPAGE™ MOPS SDS Running Buffer (Thermo Fisher Scientific). Proteins were transferred to a PVDF membrane via wet transfer in buffer containing 10% tris/glycine, 0.5% SDS, and 10% ethanol. Membranes were blocked with 3% bovine serum albumin in PBS–Tween (PBS + 0.1% Tween 20) and incubated with antibodies specifically recognizing Cas9 (Diagenode, C15200216), actin (Millipore MAB1501), and α-globin (Santa Cruz 31110) at 4°C overnight. Membranes were washed at least three times with PBS–Tween and then incubated with HRP-conjugated secondary antibodies (Santa Cruz). Protein bands were visualized using a ChemiDoc XRS imaging system (Bio-Rad).

### RP-HPLC analysis of globin chains

RP-HPLC analysis was performed using a NexeraX2 SIL-30AC chromatograph and the LC Solution software (Shimadzu). A 250 × 4.6  mm, 3.6  μm Aeris Widepore column (Phenomenex) was used to separate globin chains by HPLC. Samples were eluted with a gradient mixture of solution A (water/acetonitrile/trifluoroacetic acid, 95:5:0.1) and solution B (water/acetonitrile/trifluoroacetic acid, 5:95:0.1). The absorbance was measured at 220  nm.

### CE-HPLC analysis of hemoglobin tetramers

Cation-exchange HPLC analysis was performed using a NexeraX2 SIL-30AC chromatograph and the LC Solution software (Shimadzu). A 2 cation-exchange column (PolyCAT A, PolyLC, Columbia, MD) was used to separate hemoglobin tetramers by HPLC. Samples were eluted with a gradient mixture of solution A (20  mM Bis-Tris, 2  mM KCN, pH 6.5) and solution B (20  mM Bis-Tris, 2  mM KCN, 250  mM NaCl, pH 6.8). The absorbance was measured at 415  nm.

### Sickling assay

HSPC-derived mature RBCs, obtained at the end of the erythroid differentiation, were incubated under gradual hypoxic conditions (20% O_2_ for 20  min; 10% O_2_ for 20  min; 5% O_2_ for 20  min; 0% O_2_ for 60–120  min) and a time course analysis of sickling was performed in real time by video microscopy. Images were captured every 20  min using a spinning disk confocal microscope (Zeiss) and a 40× objective. Throughout the time course, images were captured and then processed with ImageJ to determine the percentage of sickle/non-sickle RBCs per field of acquisition in the total RBC population. Approximately 500 cells were counted per condition.

### DNA and RNA extraction

Genomic DNA was extracted using PURE LINK Genomic DNA Mini Kit (Life Technologies) or Quick-DNA/RNA Miniprep (ZYMO Research) following manufacturer’s instructions. Total RNA from HEK293T was extracted using the RNeasy Micro Kit (QIAGEn) following manufacturer’s instructions. RNA from erythroid cells was extracted using Quick-DNA/RNA Miniprep (ZYMO Research) and treated with DNase (DNase I kit; Invitrogen) according to manufacturer’s instructions.

### cDNA synthesis and RT-qPCR

Synthesis of complementary DNA (cDNA) from HEK293T samples was performed using the High-capacity cDNA reverse transcription Kit (Thermo Fisher Scientific). Mature transcripts from HSPC-derived erythroblasts were reverse-transcribed using SuperScript First-Strand Synthesis System for RT-qPCR (Invitrogen) with oligo(dT) primers. RT-qPCR was performed using primers listed in Table 2, the iTaq universal SYBR Green master mix (Bio-Rad), and the CFX384 Touch Real-Time PCR Detection System (Bio-Rad).

**Table 2.** List of primers used for RT-qPCR

Amplified region	F/R	Sequence (5′ to 3′)
HBA	F	CGGTCAACTTCAAGCTCCTAA
	R	ACAGAAGCCAGGAACTTGTC
HBB	F	GCAAGGTGAACGTGGATGAAGT
	R	TAAAGCATCAGGAGTGGACAGA
HBG1 + HBG2	F	CCTGTCCTCTGCCTCTGCC
	R	GGA TTGCCAAAACGGTCAC
HBG1/2 primary transcripts	F	TGGAGCTCTCAGCTCACTATGG
	R	TTGCAGAATAAAGCCTATCCTTGA
Cas9	F	GGACTCCCGGATGAACACTAAG
	R	GTTGTTGATCTCGCGCACTTT

### DNA methylation analysis

Genomic DNA was treated with bisulfite using EZ DNA Methylation-Gold™ Kit following manufacturer’s instructions (ZYMO Research). After bisulfite conversion, DNA was PCR-amplified using the PyroMark PCR Kit (QIAGEN) (Table 3) and then purified using the QIAquick PCR Purification Kit (QIAGEN). The PCR products were cloned in pCR4-TOPO vector following the TOPO™ TA Cloning™ Kit for sequencing protocol. The ligation products were transformed into E. coli One Shot™ TOP10 competent bacteria (Thermo Fisher Scientific). Plasmids extracted from single bacterial colonies were sequenced by Sanger sequencing and the results were analyzed using the Quantification Tools for Methylation Analysis (QUMA) tool. Alternatively, bisulfite-converted DNA was first PCR-amplified using the PyroMark PCR kit (QIAGEN) and primers containing specific DNA stretches (MR3 for forward and MR4 for reverse primers; Table 3). Amplicons were then purified using Ampure XP beads (Beckman Coulter) and used as template for a second PCR using the Phusion High-Fidelity polymerase (NEB) with primers containing Unique Dual Index (UDI) Illumina-compatible barcodes annealing to MR3 and MR4 sequences (Table 3). The amplicons were pooled equimolarly, purified using the High Pure PCR Product Purification Kit (Sigma–Aldrich), and sequenced using Illumina NovaSeq 6000 system (paired-end sequencing; 2 × 150 bp) to obtain a minimum of 100 000 reads per amplicon. NGS data were analyzed using CRISPResso2 [60], measuring the percentage of converted/unconverted cytidines in CpG nucleotides. Sequenced CpGs were aligned to hg38 reference genome, and their positions are reported in Table 4.

**Table 3.** List of primers used for PCR in bisulfite-treated samples

Primer	F/R	Sequence (5′ to 3′)
HBG	F	GTTTTGGTATTTTTTATGATGGGAG
	R	AACCTTATCCTCCTCTATAAAATAACC
HBG-NGS1	F	GCAGCGTCAGATGTGTATAAGAGACAGGTTTTGGTATTTTTTATGGTGGGAG
	R	TGGGCTCGGAGATGTGTATAAGAGACAGAATCAAAACAAAACTAACCAACCC
HBG-NGS2	F	GCAGCGTCAGATGTGTATAAGAGACAGGGGTTGGTTAGTTTTGTTTTGATT
	R	TGGGCTCGGAGATGTGTATAAGAGACAGAACCTTATCCTCCTCTATAAAATAACC
UDI-3	F	AATGATACGGCGACCACCGAGATCTACACGGCAGATCTCGTCGGCAGCGTCAGATGTG
	R	CAAGCAGAAGACGGCATACGAGATGAGCAGCGGTCTCGTGGGCTCGGAGATGT
UDI-4	F	AATGATACGGCGACCACCGAGATCTACACCTATGTTATCGTCGGCAGCGTCAGATGTG
	R	CAAGCAGAAGACGGCATACGAGATTGTTGATCGTCTCGTGGGCTCGGAGATGT
UDI-5	F	AATGATACGGCGACCACCGAGATCTACACGTTGACGCTCGTCGGCAGCGTCAGATGTG
	R	CAAGCAGAAGACGGCATACGAGATGTCCTTCGGTCTCGTGGGCTCGGAGATGT
UDI-6	F	AATGATACGGCGACCACCGAGATCTACACATCTACGATCGTCGGCAGCGTCAGATGTG
	R	CAAGCAGAAGACGGCATACGAGATCCGGCATCGTCTCGTGGGCTCGGAGATGT
UDI-7	F	AATGATACGGCGACCACCGAGATCTACACCTCGACAGTCGTCGGCAGCGTCAGATGTG
	R	CAAGCAGAAGACGGCATACGAGATCTTCGTAGGTCTCGTGGGCTCGGAGATGT
UDI-8	F	AATGATACGGCGACCACCGAGATCTACACGAGGCTGCTCGTCGGCAGCGTCAGATGTG
	R	CAAGCAGAAGACGGCATACGAGATGACGCATCGTCTCGTGGGCTCGGAGATGT
UDI-9	F	AATGATACGGCGACCACCGAGATCTACACCCTCGTAGTCGTCGGCAGCGTCAGATGTG
	R	CAAGCAGAAGACGGCATACGAGATTGCCGTAGGTCTCGTGGGCTCGGAGATGT
UDI-10	F	AATGATACGGCGACCACCGAGATCTACACCATAGGCATCGTCGGCAGCGTCAGATGTG
	R	CAAGCAGAAGACGGCATACGAGATAAGGAATCGTCTCGTGGGCTCGGAGATGT
UDI-11	F	AATGATACGGCGACCACCGAGATCTACACAGATGAACTCGTCGGCAGCGTCAGATGTG
	R	CAAGCAGAAGACGGCATACGAGATTCGAGTAGGTCTCGTGGGCTCGGAGATGT
UDI-12	F	AATGATACGGCGACCACCGAGATCTACACCCGAGTATTCGTCGGCAGCGTCAGATGTG
	R	CAAGCAGAAGACGGCATACGAGATCTCGAATCGTCTCGTGGGCTCGGAGATGT
UDI-13	F	AATGATACGGCGACCACCGAGATCTACACAATATTGATCGTCGGCAGCGTCAGATGTG
	R	CAAGCAGAAGACGGCATACGAGATCGCCGATTGTCTCGTGGGCTCGGAGATGT
UDI-14	F	AATGATACGGCGACCACCGAGATCTACACGTATACCGTCGTCGGCAGCGTCAGATGTG
	R	CAAGCAGAAGACGGCATACGAGATTCGGCGAAGTCTCGTGGGCTCGGAGATGT
UDI-15	F	AATGATACGGCGACCACCGAGATCTACACGATCCAACTCGTCGGCAGCGTCAGATGTG
	R	CAAGCAGAAGACGGCATACGAGATGAGGCCAGGTCTCGTGGGCTCGGAGATGT
UDI-16	F	AATGATACGGCGACCACCGAGATCTACACAGATACGCTCGTCGGCAGCGTCAGATGTG
	R	CAAGCAGAAGACGGCATACGAGATATAAGTTCGTCTCGTGGGCTCGGAGATGT
UDI-17	F	AATGATACGGCGACCACCGAGATCTACACGGTATCTTTCGTCGGCAGCGTCAGATGTG
	R	CAAGCAGAAGACGGCATACGAGATCCAATACGGTCTCGTGGGCTCGGAGATGT
UDI-18	F	AATGATACGGCGACCACCGAGATCTACACCCTCTGGCTCGTCGGCAGCGTCAGATGTG
	R	CAAGCAGAAGACGGCATACGAGATTGTGCTTCGTCTCGTGGGCTCGGAGATGT
UDI-19	F	AATGATACGGCGACCACCGAGATCTACACCCATTGTGTCGTCGGCAGCGTCAGATGTG
	R	CAAGCAGAAGACGGCATACGAGATGTATTAAGGTCTCGTGGGCTCGGAGATGT
UDI-23	F	AATGATACGGCGACCACCGAGATCTACACTGTCCACGTCGTCGGCAGCGTCAGATGTG
	R	CAAGCAGAAGACGGCATACGAGATCCAGATTCGTCTCGTGGGCTCGGAGATGT
UDI-24	F	AATGATACGGCGACCACCGAGATCTACACGACACACTTCGTCGGCAGCGTCAGATGTG
	R	CAAGCAGAAGACGGCATACGAGATTCAATCAGGTCTCGTGGGCTCGGAGATGT

**Table 4.** Genomic position of CpG within the *HBG* promoters

Region	CpG number	Position (hg38)
*HBG* promoters	−252	Chr11:5250109 (HBG1)
		Chr11:5255698 (HBG2)
	−162	Chr11:5250019 (HBG1)
		Chr11:5255608 (HBG2)
	−53	Chr11:5249910 (HBG1)
		Chr11:5255499 (HBG2)
	−50	Chr11:5249907 (HBG1)
		Chr11:5255496 (HBG2)
	+6	Chr11:5249851 (HBG1)
		Chr11:5255440 (HBG2)
	+17	Chr11:5249840 (HBG1)
		Chr11:5255429 (HBG2)
	+50	Chr11:5249807 (HBG1)
		Chr11:5255396 (HBG2)

### *In silico* predicted off-targets

The sgRNA-dependent off-target sites were predicted *in silico* using COSMID [61]. The following settings were used for each sgRNA: PAM suffix: NGG; number of allowed mismatches with no InDels: 3; number of allowed mismatches with 1-base Del: 2; number of allowed mismatches with 1-base Ins: 2.

### Whole transcriptome sequencing

Total RNA was isolated using the Quick-DNA/RNA Miniprep Kit (Zymo), including a DNase treatment step. RNA quality was assessed by capillary electrophoresis using High Sensitivity RNA reagents with the Fragment Analyzer (Agilent Technologies) and the RNA concentration was measured by using both Xpose spectrophotometry (Trinean) and Fragment Analyzer (Agilent Technologies) capillary electrophoresis. RNA sequencing (RNA-seq) libraries were prepared starting from 100 ng of total RNA using the RNA Library Prep Kits with Polaris Depletion (Watchmaker) as recommended by the manufacturer to deplete ribosomal RNA and globin mRNAs. qPCR, according to the Illumina qPCR Quantification Protocol Guide was performed to quantify the RNA-seq libraries. An equimolar pool of the final indexed RNA-Seq libraries was prepared and sequenced using the Illumina NovaSeq X system (paired-end sequencing; 2 × 100 bp, 50 million reads per library).

Read quality was assessed using FastQC [version 0.11.9; https://www.bioinformatics.babraham.ac.uk/projects/fastqc/]. Adapter sequences and low-quality bases (Q < 20) were trimmed from raw reads with BBDuk [version 38.92; https://sourceforge.net/projects/bbmap/]; moreover, the first 10 nucleotides were force-trimmed for low quality. Reads shorter than 35 bp post-trimming were discarded. Trimmed reads were aligned to the human reference genome (hg38) using STAR [version 2.7.9a] [62]. Raw gene counts were generated in R-4.1.1 using the *featureCounts* function of the *Rsubread* package [version 2.8.1] [63] and the GENCODE 44 basic gene annotation for hg38 reference genome. Raw gene counts were normalized to counts per million mapped reads (CPM) and to fragments per kilobase of exon per million mapped reads (FPKM) using the *edgeR* R package [version 3.36.0] [64]; only genes with a CPM greater than 1 in at least 3 samples were retained for differential analysis. Differential gene expression analysis was performed using the *glmQLFTest* function (using donor as a blocking variable for SCD samples) or the *exactTest* function (for HD samples) of the *edgeR* R package. Genes with FDR < 0.05 and absolute log_2_ fold change ≥ 1 were defined as differentially expressed. Functional enrichment analysis on MsigDB Hallmark gene sets was performed using the *enricher* function of the *clusterProfiler* R package [version 4.12.5] [65]. To investigate the expression profile of transposable elements, we used the TEtranscripts package (ADD REF). First, trimmed reads were aligned to the human reference genome (hg38) using STAR [version 2.7.9a] [62], setting winAnchorMultimapNmax to 200 and outFilterMultimapNmax to 100, as recommended by TEtranscripts authors. The raw count matrix for transposable elements was obtained by the TEcount function of TEtranscripts package, using the hg38 GTF files for TE annotation provided on the TEtranscripts website (https://www.mghlab.org/software/tetranscripts). Raw counts for transposable elements were collapsed by families and groups and normalized to CPM using the edgeR R package [version 3.36.0] [64] within R-4.1.1. Differential gene expression analysis was performed using the glmQLFTest function (using donor as a blocking variable for SCD samples) or the exactTest function (for HD samples) of the edgeR R package.

### Nanopore-based genomic DNA sequencing and combined methylation/hydroxymethylation analysis

Genomic DNA (0.8 μg) was fragmented using a Covaris g-TUBE to achieve an average fragment size of 8–9 kb. The DNA was brought to a final volume of 50 μl and centrifuged at 5500 × *g* for 1 min. The tube was then inverted, and the centrifugation was repeated for an additional 1 min. The sheared DNA fragments were processed using the SQK-LSK114 library preparation kit protocol from Oxford Nanopore Technologies. This included end repair, A-tailing of the DNA, and adapter ligation. Libraries were individually sequenced on a PromethION P2i device, using one flow cell (FLO-PRO114M) per sample to achieve an average coverage of 30×. Raw Pod5 data were basecalled using MinKNOW to detect 5mCpG and 5hmCpG modifications. The resulting reads were aligned to the CHM13-T2T and GRCh38.p14 annotated human genome assemblies using Minimap2, generating BAM files. Methylation and hydroxymethylation data were extracted and visualized using Modbamtools and Methylartist, enabling both global analyses and locus-specific investigations. For the analysis of repetitive elements, the BED files containing the locations of SINE and LINE transposable elements were retrieved from RepeatMasker.org. The analysis was then performed using Bedtools for the intersection of regions of interest and Modkit for the annotation and quantification of associated epigenetic modifications.

### Xenotransplantation of HSPCs in NBSGW mice

NOD.Cg-KitW-41JTyr + PrkdcscidIl2rgtm1Wjl/ThomJ (NBSGW) mice were housed in a pathogen-free facility. Control or edited mobilized CD34^+^ cells (3 × 10^5^ cells per mouse) were transplanted into non-irradiated NBSGW male or female mice of 6–9 weeks of age via retro-orbital sinus injection. NBSGW mice were conditioned with busulfan (Sigma–Aldrich) injected intraperitoneally (15 mg/kg body weight) 24 h before transplantation. Sixteen weeks after transplantation, NBSGW primary recipients were euthanized. Cells were harvested from BM, thymus, spleen, and blood and stained with antibodies against the following murine and human surface markers: murine CD45 (1/50 mCD45-VioBlue; Miltenyi Biotec), human CD45 (1/50 hCD45-APCvio770; Miltenyi Biotec), human CD3 (1/50 CD3-APC; Miltenyi Biotec), human CD14 (1/50 CD14-PECy7; BD Biosciences), human CD15 (1/50 CD15-PE; Miltenyi Biotec), human CD11b (1/100 CD11b-APC; Miltenyi Biotec), human CD19 (1/100 CD19-BV510; BD Biosciences), human CD235a (1/50 CD235a-PE; BD Biosciences), human CD71 (1/10 CD71-APC; BD Biosciences), CD36 (1/50 CD36-FITC; BD Biosciences), and CD34 (1/100 CD34-PE-Vio770; Miltenyi Biotec). Cells were analyzed by flow cytometry using the Novocyte analyzer (Agilent) and the FlowJo software (BD Biosciences). Human BM CD45^+^ cells were sorted by immunomagnetic selection with CD45 MicroBeads (Miltenyi Biotec) and subjected to immunostaining with biotinylated antibodies that recognized the following surface markers: CD3 (dilution 1/25, clone HIT3a; BD), CD19 (dilution 1/25, clone HIB19; BD), B220 (dilution 1/50, clone RA3-6B2; BD), Ter119 (dilution 1/50, clone TER-119; BD), and mCD117 (clone 2B8; BD). BM cells were washed and incubated with 20 μl of Anti-Biotin beads (Miltenyi Biotec). After washing, the cells were magnetically purified using an LS column (Miltenyi Biotec) according to the manufacturer’s instructions. Cells from the negative fraction were immunostained with the following antibodies: CD235a-PE (dilution 1/5000, BD) and hCD45-BV510 (dilution 1/100, BD). The hCD45^low/−^/CD235a^high^ cells were sorted using the MA900 cell sorter (Sony Biotechnology, San Jose, CA) and subjected to DNA methylation, RT-qPCR, RP-HPLC, and CE-HPLC analyses. Flow cytometry analysis for HbF was performed as previously described on cells from the negative fraction. All experiments and procedures were performed in compliance with the French Ministry of Agriculture’s regulations on animal experiments and were approved by the regional Animal Care and Use Committee (APAFIS#2019061312202425_v4). Mice were housed in a temperature-(20°C-22°C) and humidity (40%–50%)-controlled environment with 12:12 h light–dark cycle and fed *ad libitum* a standard diet.

### ChIP-seq data source

We analyzed publicly available H3K4me3, H3K27ac, H3K9ac, H3K27me3, and H3K9me3 ChIP-seq datasets in human primary fetal, adult, and HEK293T [66, 67]. For the histone modifications in human primary fetal and adult, we downloaded the hg18-mapped read bed files from Gene Expression Omnibus (GSM908038, GSM908039, GSM908050, GSM908051, GSM908048, GSM908049, GSM908042, GSM908043, GSM908040, GSM908041) and converted them to the hg19 build of the human genome using the UCSC liftOver tool. We used MACS213 to build signal tracks and call peaks. For HEK293T dataset, we downloaded the hg19-mapped read bed files from Gene Expression Omnibus (GSM4301071, GSM4301076, GSM4301082, GSM4301086).

### Statistics and reproducibility

No statistical method was used to predetermine the sample size. We used the minimum number of replicates (*n*  = 3) to perform statistics. Biologically independent experiments reported here are from independent (i) splits of each cell type, (ii) primary cells from different donors, or (iii) mice. No data were excluded from the analyses. The experiments were not randomized. The investigators were not blinded to allocation during experiments and outcome assessment. Statistical analyses performed are reported in figure legends and were performed with Prism version 10.

## Results

### Different epigenetic marks characterize the *HBG* promoters in adult versus fetal primary erythroblasts

**Figure 1.**
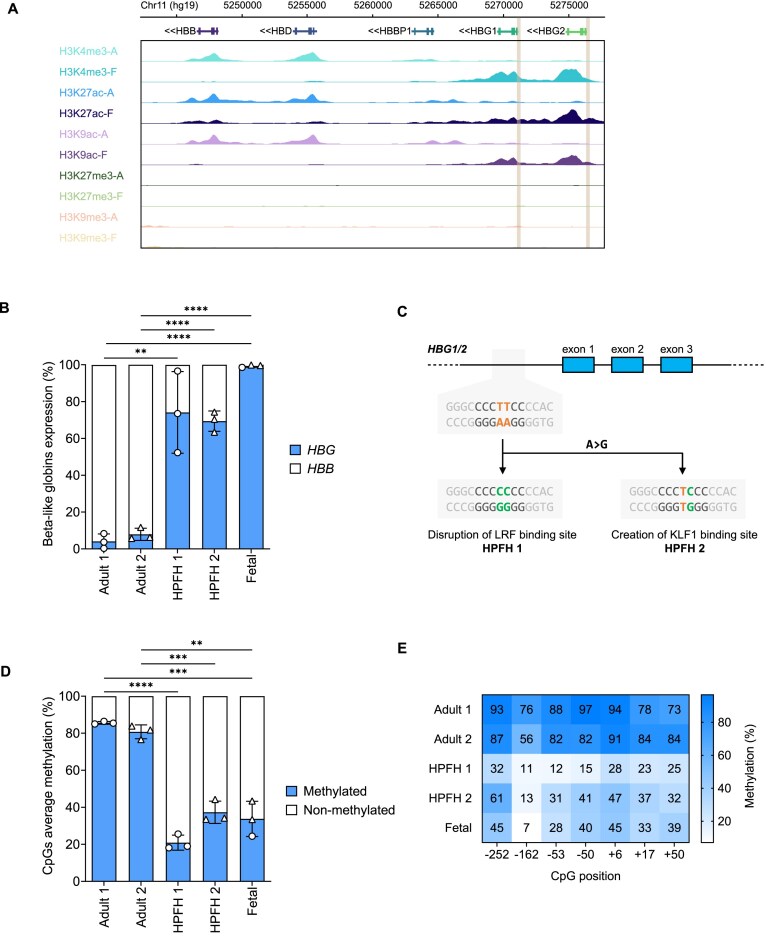
Epigenetic status of the *HBG* promoters in adult and fetal erythroid cells. (**A**) ChIP-seq analysis of *HBG1/2* promoters (highlighted). Epigenetic modifications include H3K27 acetylation (H3K27ac), H3K4 trimethylation (H3K4me3), H3K9 acetylation (H3K9ac), H3K27 trimethylation (H3K27me3), and H3K9 trimethylation (H3K9me3) in adult (A) and fetal erythroblasts (F). (**B**) Percentage of β-like globin mRNA (*HBG* and *HBB*) expression measured by RT-qPCR in adult erythroblasts at day 6 of the erythroid differentiation (Adult 1), adult erythroblasts at day 13 of the erythroid differentiation (Adult 2), edited adult erythroblasts harboring the disrupted LRF binding site (HPFH 1), edited adult erythroblasts harboring the KLF1 binding site (HPFH 2), and fetal erythroblasts (Fetal) at day 6 (circle) or day 13 (triangle) of erythroid differentiation. Of note, for 2 of the 3 fetal liver donors, we collected cells at day 13 since at day 6 the differentiation toward the erythroid lineage was delayed compared to the first donor and a relatively large fraction of the cells was not committed toward the erythroid lineage. β-like globin expression was normalized to *HBA*. (**C**) Schematic representation of the *HBG* gene on chromosome 11. The sequence corresponding to the LRF binding site, before and after base editing, is reported. (**D**) Average methylation of CpGs within the *HBG* promoters by bisulfite sequencing. (**E**) Methylation analysis of individual CpGs. All data are expressed as mean ± SD from 4 biologically independent experiments (3 adult donors for Adult 1 and HPFH 1, 3 adult donors for Adult 2 and HPFH 2, 1 fetal donor at day 6, and 2 fetal donors at day 13). Asterisks indicate level of statistical significance: **P ≤ .01; ***P ≤ .001; ****P ≤ .0001; no asterisk = not significant (unpaired *t*-test).

To provide a comprehensive characterization of the epigenetic landscape associated with the *HBG* promoters, we reanalyzed publicly available ChIP sequencing data comparing adult and fetal HSPC-derived erythroblasts [68]. The levels of histone marks typically associated with active transcription, such as histone 3 lysine 4 trimethylation (H3K4me3), histone 3 lysine 27 acetylation (H3K27ac), and histone 3 lysine 9 acetylation (H3K9ac), were high in fetal erythroblasts, while these modifications were absent in adult erythroblasts that express low levels of γ-globin (Fig. 1A). On the contrary, repressive histone modifications (H3K27me3 and H3K9me3) were absent in both adult and fetal cells (Fig. 1A). These data confirmed the positive correlation between active histone marks, the activity of the *HBG* promoters, and γ-globin expression. We then investigated DNA methylation in erythroid cells derived from adult or fetal liver HSPCs (expressing mainly *HBB* and *HBG*, respectively; Fig. 1B), or from HSPCs treated with base editors to generate HPFH and HPFH-like mutations disrupting the binding site for the LRF transcriptional repressor (HPFH1) or creating a binding site for the KLF1 transcriptional activator (HPFH2) in the *HBG* promoters [39] (Fig. 1C and Supplementary Fig. S1A–F). These edited fetal-like cells showed increased levels of γ-globin compared to the control cells (up to ∼70% of the total β-like globin mRNAs) (Fig. 1B). Adult cells expressing low *HBG* levels displayed a general hypermethylation of all CpGs within the *HBG* promoters. On the contrary, edited fetal-like cells and fetal erythroblasts (characterized by high *HBG* expression) showed substantially reduced CpG methylation levels (Fig. 1D and E). These results show a positive correlation between *HBG* gene activity and DNA demethylation. Furthermore, these data suggest the CpG methylation is a highly dynamic process that can be modulated to achieve HBG gene activation.

### Targeted histone acetylation induces *HBG* activation in HEK293T

We developed an epigenome editing strategy to investigate whether histone acetylation and DNA methylation individually have a direct role in *HBG* expression and if they can be modulated to reactivate HbF using epigenome editors (dCas9-CBP to deposit histone acetylation and Tet1-dCas9 to demethylate DNA). Previous studies demonstrated that, at least in the context of a repressive epigenome editing system, simultaneous modulation of DNA methylation and histone modifications is crucial for ensuring the persistence of epigenetic modifications [52]. Thus, we also combined deposition of histone acetylation with DNA demethylation to recreate an epigenetic landscape similar to that observed in fetal cells at the *HBG* promoters and achieve stable *HBG* activation.

First, plasmids coding for the epigenome editor dCas9-CBP core (referred hereafter as CBP) and the sgRNAs targeting the *HBG* promoters (g-28, g-115, g-165, g-197, g-211) [35, 56] (Supplementary Fig. S2A) were co-transfected in the HEK293T cell line.

Of note, the CBP core contains the acetyltransferase domain but not the transactivation domain, indicating that gene activation is due specifically to the acetyltransferase activity [56].

Three days after transfection, we evaluated *HBG* expression by RT-qPCR. Control cells transfected with either the dCas9 or CBP plasmid alone, and those transfected with dCas9 and four sgRNAs, showed negligible *HBG* expression. Conversely, cells treated with CBP and individual sgRNAs exhibited a strong increase in γ-globin transcription, reaching >10^3^-fold increase in *HBG* expression (compared to dCas9 only), except for g-165, which led to low gene activation (Supplementary Fig. S2B). Interestingly, this activity was cumulative, as an even higher activation (up to 10^4^-fold change) was observed using four sgRNAs, with the most effective combination being g-28, -115, -197, and -211 (referred hereafter as 4x sgRNAs) (Supplementary Fig. S2B). Next, we tested the long-term efficacy of our strategy by maintaining the transfected cells in culture for 17 days and analyzing *HBG* expression at different time points. In addition to CBP, we tested Tet1-dCas9 (referred hereafter as Tet1), which is capable of removing DNA methylation. Although high *HBG* expression was initially observed in cells treated with either CBP alone or in combination with Tet1 (using either the individual best-performing sgRNAs or the optimal 4x sgRNAs combination), *HBG* reactivation was not sustained over time, eventually returning to baseline levels at the final time point (Supplementary Fig. S2C–E). Moreover, Tet1 alone was not able to reactivate *HBG* expression neither 3 days post-transfection nor at later timepoints (Supplementary Fig. S2C–E). To investigate the failed *HBG* reactivation observed with Tet1, we analyzed the methylation status of the CpGs in the *HBG* promoters in the HEK293T cell line. Although this cell line does not express γ-globin, all the CpGs were poorly methylated, suggesting that DNA methylation does not play a role in regulating *HBG* expression in HEK293T cells (Supplementary Fig. S2F). As expected, in HEK293T the β-globin locus presents no active histone marks (H3K4me3, H3K27ac) but a general heterochromatin state characterized by H3K27 trimethylation that is consistent with a Polycomb-mediated globin gene repression (Supplementary Fig. S2G).

Overall, these results showed that CBP-mediated histone acetylation is a robust approach to induce *HBG* gene transcription in HEK293T and the combination of four sgRNAs results in a more potent gene activation compared to the individual sgRNAs. Nevertheless, CBP is not sufficient to obtain long-term activation in this cell model. Furthermore, HEK293T are not the appropriate cellular model to evaluate Tet1-dCas9 effect on reactivation of γ-globin genes and to provide insights on the stability of gene activation obtained when combining DNA and histone epigenetic modifications.

### Removal of DNA methylation and deposition of histone acetylation reactivates HbF and corrects the sickling cell phenotype in the erythroid progeny of HSPCs

**Figure 2.**
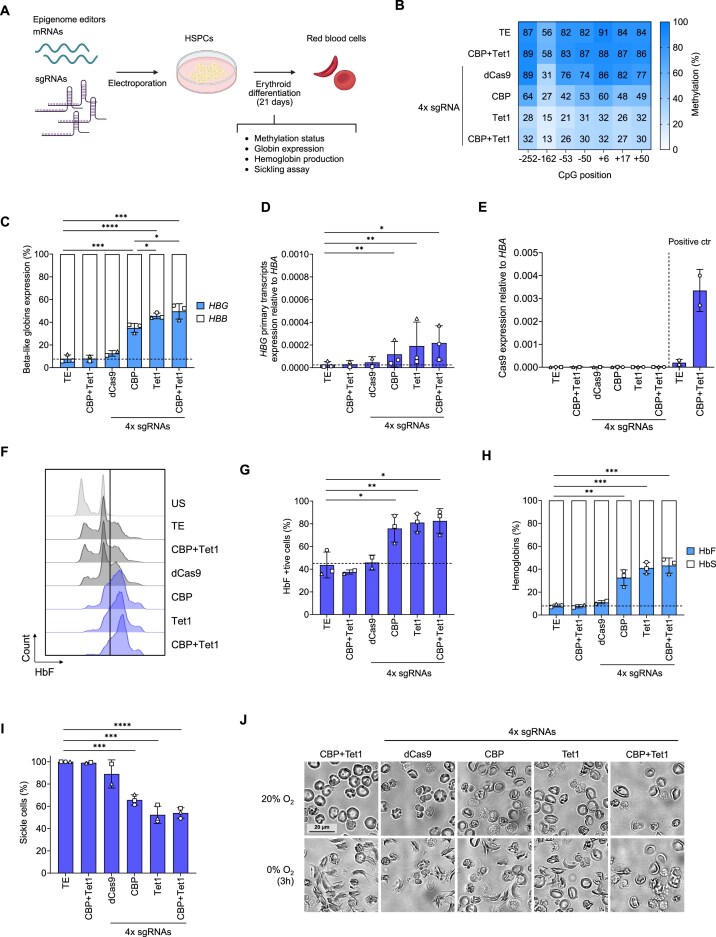
HbF reactivation in the erythroid progeny of SCD HSPCs treated with epigenome editors. (**A**) Scheme of the experimental procedure used for experiments in non-mobilized or BM-derived SCD HSPCs. (**B**) Methylation analysis of CpGs within the *HBG* promoters by bisulfite sequencing. Cells electroporated with sgRNAs and mRNA expressing the editors were analyzed at day 13 of the erythroid differentiation. (**C**) Percentage of β-like globins (*HBG* and *HBB*) expression normalized to *HBA* expression measured by RT-qPCR at day 13 of the erythroid differentiation. (**D**) Fold-change increase of *HBG* primary transcripts normalized to *HBA* expression measured by RT-qPCR. (**E**) Cas9 expression normalized to *HBA* expression measured by RT-qPCR at day 13 of erythroid differentiation. HSPCs (*n* = 2) electroporated with TE buffer or editors collected and analyzed by RT-qPCR 3 days after transfection served as positive controls. (**F**) Histogram plot of HbF-positive RBCs measured by flow cytometry at day 20 of the erythroid differentiation. The two peaks in the HbF-negative population might derive from the different autofluorescence of enucleated and nucleated cell populations. (**G**) Percentage of HbF-positive RBCs measured by flow cytometry. (**H**) HbF and HbS levels measured by CE-HPLC in RBCs at day 19 of the erythroid differentiation. The percentage of each Hb type was calculated over the total Hb tetramers. (**I**) Frequency of sickle RBCs measured 3 h after O_2_ deprivation (normalized to the TE controls). (**J**) Representative pictures of RBCs at 20% O_2_ and after 3 h at 0% O_2_. All data are expressed as means ± standard deviation (SD) from 3 independent experiments. Each symbol represents a different donor. Asterisks indicate level of statistical significance: *P ≤ .05; **P ≤ .01; ***P ≤ .001; ****P ≤ .0001; no asterisk = not significant (unpaired *t*-test).

We reasoned that additional studies using primary cells carrying hypermethylated γ-globin promoters were needed to assess the long-term effects of DNA demethylation and histone acetylation on γ-globin expression. To this aim, we delivered the editing reagents as RNA in HSPCs obtained from patients with SCD. Cells were electroporated with CBP and Tet1 editors alone or in combination with 4x sgRNAs. The dCas9 with no effector but with 4x sgRNAs was used as an additional control. After electroporation, cells were differentiated toward the erythroid lineage (Fig. 2A). No significant differences were observed in the expression of early and late erythroid markers or in the enucleation rate between control and treated cells, showing no impact of the editing procedure on erythroid differentiation (Supplementary Fig. S3A–E and S1A). While control primary erythroblasts showed highly methylated CpGs within the *HBG* promoters, epigenome editors caused a general demethylation with Tet1 and CBP + Tet1 being more effective than CBP alone (Fig. 2B and Supplementary Fig. S3F). DNA methylation levels were inversely correlated with γ-globin mRNA expression (measured by RT-qPCR) accounting for up to 40% of the total β-like globin transcripts in Tet1- and CBP + Tet1-treated samples (Fig. 2C). The analysis of primary transcripts confirmed an active transcription of *HBG* genes in treated cells (Fig. 2D) even in the absence of detectable epigenome editor expression, suggesting durable *HBG* gene activation (Fig. 2E and Supplementary Fig. S3G). Flow cytometry revealed 80% of HbF-positive RBCs in all the edited samples (Fig. 2F–G and Supplementary Fig. S3H and I). HbF reactivation was also observed by cation-exchange HPLC (CE-HPLC) with 30% of HbF tetramers in samples treated with CBP and >40% HbF in Tet1- and CBP + Tet1-treated cells. Concomitantly, HbS levels were substantially reduced (Fig. 2H). Reverse-phase HPLC (RP-HPLC) analysis of individual globin chains confirmed γ-globin reactivation and β-globin down-regulation with normal α/non-α globin chain ratio (Supplementary Fig. S3J). These results demonstrate reactivation of γ-globin associated with reduced β-globin synthesis. Finally, to evaluate the correction of the sickling phenotype, RBCs were incubated under hypoxic conditions to induce HbS polymerization. After 3 h, we observed a 40% reduction in the frequency of sickle cells in CBP-treated samples and 50% in the Tet1 and CBP + Tet1 conditions, reaching levels similar to those observed in asymptomatic carriers (Fig. 2I and J).

Overall, these findings demonstrate that DNA methylation plays a direct role in HbF silencing, which can be reverted using epigenetic modifiers. This might represent a safe and efficacious strategy for β-hemoglobinopathies, such as SCD.

### Persistence of HbF expression after treatment with epigenome editors

**Figure 3.**
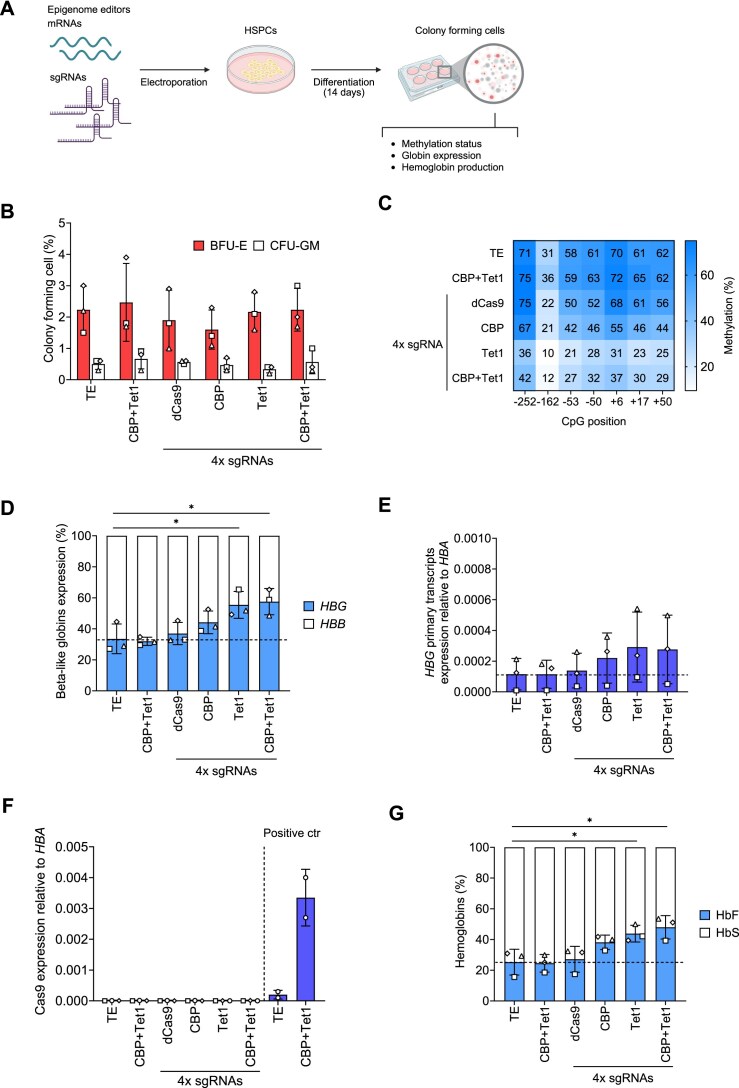
Progenitor counts and γ-globin reactivation in BFU-Es after epigenome editing. (**A**) Scheme of the experimental procedure used for experiments in BM-derived SCD HSPCs. (**B**) CFC frequency in control and edited samples. (**C**) Methylation analysis of CpGs within the *HBG* promoters by bisulfite sequencing in pools of BFU-E colonies (>25 colonies obtained in 3 independent experiments). (**D**) Percentage of β-like globins (*HBG* and *HBB*) expression normalized to *HBA* expression measured by RT-qPCR. (**E**) Fold-change increase of *HBG* primary transcripts normalized to *HBA* expression measured by RT-qPCR. (**F**) Cas9 expression normalized to *HBA* expression measured by RT-qPCR. HSPCs (*n* = 2) electroporated with TE buffer or editors were collected and analyzed by RT-qPCR 3 days after transfection and served as positive controls [same data as in panel (E)]. (**G**) HbF and HbS levels measured by CE-HPLC in BFU-Es. The percentage of each Hb type was calculated over the total Hb tetramers. All data are expressed as means ± SD from 3 independent experiments. Each symbol represents a different donor. Asterisks indicate level of statistical significance; *P ≤ .05; no asterisk = not significant (unpaired *t*-test).

To assess the safety of the approach and to measure HbF production in cells that underwent a higher number of cell divisions compared to erythroblasts in liquid culture, HSPCs from SCD patients were plated in a semi-solid medium that allows the clonal growth and differentiation of erythroid and granulocyte/monocyte progenitors (colony-forming cell, CFC assay) (Fig. 3A). The frequency of erythroid (BFU-E) colonies and granulocyte/monocyte (CFU-GM) colonies was similar in all the treated conditions and comparable to the controls, demonstrating no impact of epigenome editing on hematopoietic progenitors (Fig. 3B). As observed in liquid cultures, pools of BFU-E colonies treated with editors and sgRNAs presented a general hypomethylation in all the CpGs of the *HBG1/2* genes (Fig. 3C and Supplementary Fig. S4A). These results correlated with the increase of γ-globin transcripts in treated samples (even when epigenome editors are no longer expressed), with Tet1 and CBP + Tet1 being more potent than CBP alone (Fig. 3D–F). Finally, all the treated cells showed HbF reactivation (as measured by CE-HPLC), which was more pronounced in Tet1 and CBP + Tet1 samples (Fig. 3G). We performed an additional CFC assay with HSPCs from two healthy individuals (giving rise to a higher number of colonies compared to SCD patients’ HSPCs) [69, 70]. We observed no morphological changes across the different conditions in the colonies, while still detecting HbF reactivation (Supplementary Fig. S4B–E).

### Specificity profile of epigenome editors targeting *HBG*

To evaluate the occurrence of unintended outcomes at the RNA level (e.g. due to off-target activity of epigenome editors), we analyzed the transcriptome of control and edited HSPCs derived from 3 different SCD donors 72 h after electroporation.

RT-qPCR analysis showed increased *HBG* expression in HSPCs treated with CBP + Tet1-4xsgRNAs, which was less pronounced in Tet1-4xsgRNAs samples, suggesting that *HBG* reactivation after Tet1-mediated DNA demethylation alone necessitates other factors to be expressed upon erythroid differentiation (Supplementary Fig. S5A). Accordingly, erythroid populations derived from edited HSPCs showed similar frequencies of HbF-expressing cells (Supplementary Fig. S5B).

**Figure 4.**
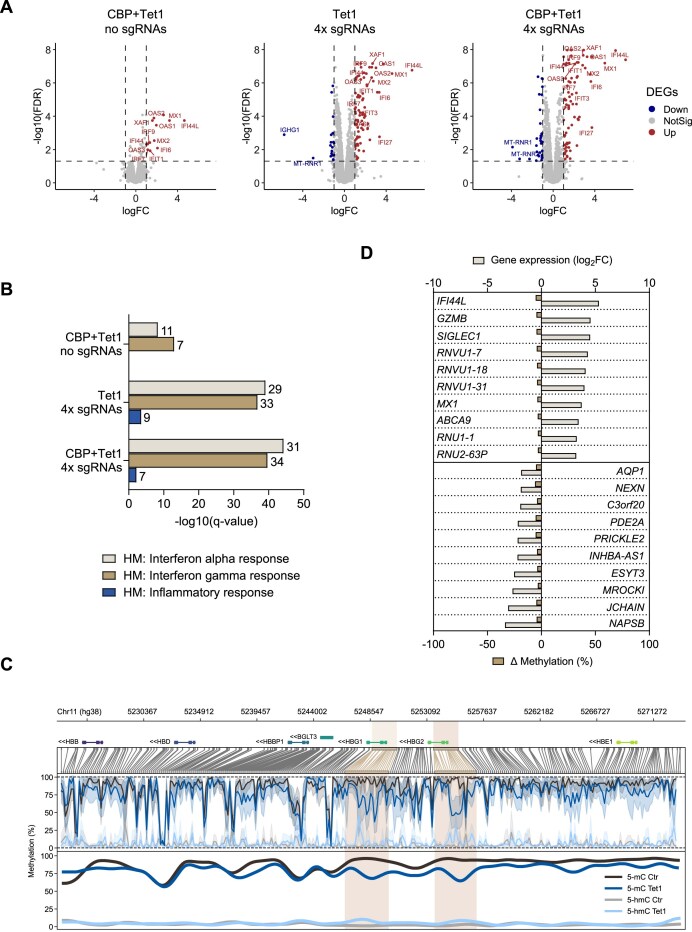
Transcriptome and DNA methylome profiles of epigenome-edited HSPCs. (**A**) Volcano plots showing differential gene expression between cells treated with CBP + Tet1 alone (left), Tet1-4xsgRNAs (middle), and CBP + Tet1-4xsgRNAs (right) and control cells. RNA-seq was performed 72 h after electroporation in 3 different donors with SCD. The horizontal dashed line indicates the threshold on the FDR  ≤0.05, and the vertical dashed lines correspond to the threshold on log_2_FC  ≥1 or ≤−1. Upregulated genes are indicated in red and downregulated genes are in blue. Genes in gray are not differentially expressed. (**B**) Gene set enrichment analyses on Hallmark gene sets from MSigDB were performed on up-regulated DEG. The enriched Hallmark gene sets are reported. We reported the number of up-regulated genes belonging to each gene set. HM, hallmark. (**C**) CpG methylation profiles in a ± 25-kb genomic region centered on the TSS of *HBG1*. Individual dots indicate the average methylation of each CpG. Linear regression was applied to the smoothed curve (bottom panel) representing the percentage of methylation levels across the 50-kb locus encompassing *HBG1* and *HBG2*. (**D**) Correlation between log_2_FC in gene expression of the top 10 up- and downregulated genes and their variation in DNA methylation levels. We calculated the average DNA methylation in a ± 25-kb genomic region centered on the TSS of DEGs in control versus treated cells.

We then compared by RNA-seq mock-electroporated HSPCs and HSPCs treated with CBP + Tet1 alone, Tet1-4xsgRNAs, or CBP + Tet1-4xsgRNAs. Cells that received only editor mRNAs showed 14 upregulated genes and no downregulated genes [log_2_ fold change (log_2_FC)  ≥ 1 or ≤−1, respectively, false discovery rate (FDR)  ≤ 0.05]. In HSPCs electroporated with Tet1 or CBP + Tet1 and the 4x sgRNAs, we observed 101 and 113 differentially expressed genes (DEGs; 81 and 80 upregulated, 20 and 33 downregulated), respectively (Fig. 4A and Supplementary Table S1). Most of the upregulated genes were in common between the different conditions (Supplementary Fig. S5C) and were involved in RNA sensing and response to viruses (e.g. IFI44L, IFI44, IFI6, IRF7, IRF9, and OAS1-3). In fact, a functional enrichment analysis focusing on MSigDB Hallmark gene sets confirmed an enrichment in genes associated with interferon alpha and gamma pathways (Fig. 4B). Accordingly, the FC of DEGs increased with the amount of electroporated RNA (Fig. 4A). Importantly, we did not observe dysregulation of genes involved in HbF regulation and DNA damage response or *in silico* predicted sgRNA-dependent off-target genes (Supplementary Fig. S5D, Fig. 4A, and Supplementary Tables S1 and S2). Finally, we observed no changes in the expression profile of transposable elements (such as LINE and SINE, which can be sensitive to DNA demethylation) in the different edited conditions compared to the controls (Supplementary Fig. S5G and Supplementary Table S7).

To further assess the specificity of our strategy, we analyzed the native DNA methylome using direct genomic DNA sequencing with Oxford Nanopore Technology in healthy donor (HD)-derived HSPCs treated with Tet1-4xsgRNAs. Focusing on a 50-kb region centered on the *HBG1/2* genes, we detected a decrease in 5-mC restricted to the *HBG* promoter regions in treated cells compared to controls (Fig. 4C and Supplementary Fig. S5E). Specifically, we quantified a 38% DNA methylation decrease at the *HBG1/2* promoters, while only −7% in the surrounding regions. In parallel, 5-hmC levels were increased, confirming that this mark is positively correlated with γ-globin expression, as observed in a nonhuman primate baboon model [18].

We then examined the variation in DNA methylation levels in a ± 25-kb region surrounding the TSS of each DEG identified via RNA-seq (Supplementary Fig. S5F and Supplementary Table S3). Our data revealed only a modest decrease in the average DNA methylation (0 to −7%) in treated cells, observed across both upregulated and downregulated genes (Fig. 4D and Supplementary Table S4). In treated cells, we analyzed ∼17 000 genes with unchanged expression levels and identified a subset of genes (400 with reduced DNA methylation and 64 with increased methylation) that exhibited changes in DNA methylation, which were not associated with gene dysregulation (Supplementary Table S5). Finally, *in silico* predicted sgRNA-dependent off-targets showed a modest change in DNA methylation (from −7% to +10%) that did not influence gene expression (Supplementary Table S6). Finally, we found no differences in DNA methylation levels at repetitive elements in control versus treated cells (Supplementary Fig. S5H and Supplementary Table S7). Altogether, these results show a modest transcriptional response related to cellular sensing to RNA and highlight the specificity of our DNA DSB-free strategy.

### Epigenome editing in repopulating hematopoietic stem cells

**Figure 5.**
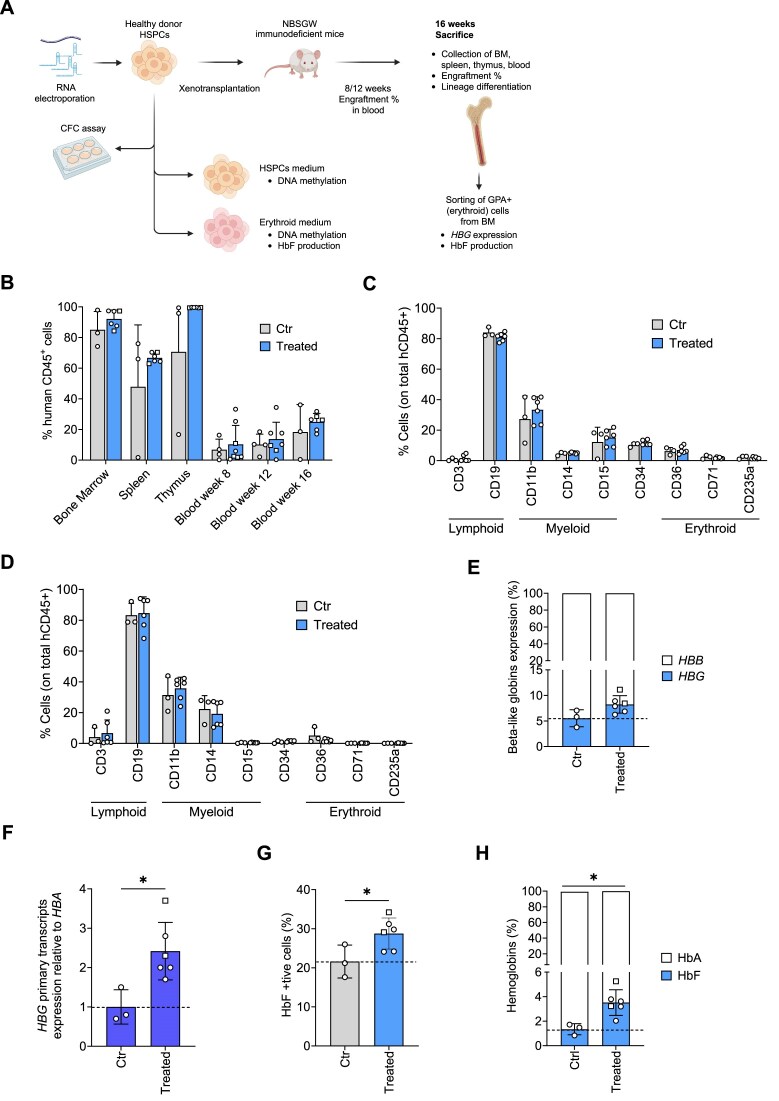
Engraftment and multi-lineage differentiation of epigenome-edited HSPCs and HbF expression in their erythroid progeny. (**A**) Scheme of the experimental procedure used for experiments of HD HSPC xenotransplantation. Epigenome editor mRNAs and sgRNAs were co-transfected in HD HSPCs. Control and edited cells were xenotransplanted into NBSGW immunodeficient mice 1 day after transfection. Mice were euthanized 16 weeks after transplantation and their hematopoietic tissues and organs were collected and analyzed. (**B**) Engraftment of human cells in NBSGW mice transplanted with control (mock-transfected) or treated HSPCs 16 weeks post-transplantation (*n* = 3 ctr group, *n*= 6 treated group). Chimerism is calculated as the percentage of human CD45^+^ cells in the total murine and human CD45^+^ cell population in BM, spleen, thymus, and peripheral blood. (**C**) Frequency of human T (CD3) and B (CD19) lymphoid, myeloid (CD11b, CD14, and CD15), erythroid (CD235, CD36, and CD71) cells, and HSPCs (CD34) in BM and (**D**) spleen of mice transplanted with control and edited HSPCs 16 weeks after the transplantation (*n* = 3 ctr group, *n*= 6 treated group). (**E**) Percentage of β-like globin (*HBG* and *HBB*) mRNAs normalized to *HBA* expression and (**F**) fold-change increase of *HBG* primary transcripts normalized to *HBA* expression measured by RT-qPCR in BM cells sorted for CD235a. (**G**) Percentage of HbF-positive cells measured by flow cytometry in BM-derived human CD235a^+^ cells. (**H**) HbF and HbA levels measured by CE-HPLC in human CD235a^+^ BM cells. The percentage of each Hb type was calculated over the total Hb tetramers. All data are expressed as mean ± SD. Each point represents an individual mouse; in the treated group squares indicate samples treated with Tet1 and circles depict samples treated with CBP + Tet1. Asterisks indicate level of statistical significance; *P ≤ .05; no asterisk = not significant (unpaired *t*-test).

To evaluate the capability of edited HSPCs to engraft and differentiate *in vivo* and test the long-term effects of epigenome editors, we transplanted human HSPCs edited with Tet1 or CBP + Tet1 into immunodeficient NBSGW mice (Fig. 5A). The *HBG* promoters were hypermethylated in control input cells, while a diminished methylation was detected in treated samples (Supplementary Fig. S6A). Erythroid cells differentiated *in vitro* from edited HSPCs showed high levels of HbF expression (Supplementary Fig. S6B). Moreover, similar progenitor frequencies were observed in the different conditions, whereas only edited BFU-E presented an increased production of HbF and γ-globin chains (Supplementary Fig. S6C–E). A clonal analysis revealed that DNA methylation was significantly reduced in Tet1- and Tet1 + CBP-treated BFU-E, and this was accompanied by a substantial increase in *HBG* expression (Supplementary Fig. S6F–G).

Sixteen weeks after transplantation, we measured the frequency of human CD45^+^ cells and the proportion of the different human lineages in the hematopoietic tissues. We observed no differences between control and treated animals in the chimerism (measured as percentage of human CD45^+^ cells on the total human and murine hematopoietic CD45^+^ cells) in the BM, spleen, thymus, and blood, and in the frequency of lymphoid, myeloid, and erythroid cells or CD34^+^ HSPCs (Fig. 5B–D). These results indicate that epigenome editing does not affect engraftment and differentiation potential of hematopoietic stem cells (HSCs).

Importantly, CD235a^+^ erythroid cells sorted from BM of animals treated with epigenome editors showed increased *HBG* mRNA and primary transcripts (Fig. 5E and F). Finally, we observed a higher percentage of HbF^+^ cells by flow cytometry (Fig. 5G) and an increase in HbF production in edited cells compared to the control group (Fig. 5H).

## Discussion

In this study, we investigated the role of DNA methylation and histone acetylation on the *HBG* promoter activity to identify the epigenetic determinants of *HBG* regulation. Analysis of epigenetic marks at the *HBG* promoters revealed a distinct pattern of modifications in adult versus fetal erythroid cells. First, we confirmed that *HBG* promoters are highly methylated in adult cells compared to fetal liver-derived erythroblasts. We then investigated the methylation pattern of the *HBG* promoters in adult erythroid cells before and after introduction of HPFH-like mutations via base editing [39]. Upon genetic modification, we observed *HBG* reactivation that was associated with DNA demethylation of the *HBG* promoters, suggesting that DNA methylation might play a role in *HBG* repression and might be modulated to reactivate HbF. In parallel, the analysis of adult and fetal histone modifications at the *HBG* promoters allowed us to identify critical histone marks (e.g. H3K27ac) characterizing active γ-globin genes. On the contrary, repressive histone marks were not associated with *HBG* silencing, suggesting that DNA methylation might play a primary role in *HBG* repression.

Nevertheless, these analyses did not allow us to determine whether the introduction of epigenetic modifications (i.e. DNA demethylation and histone acetylation) is sufficient for reactivating γ-globin expression. Thus, we further dissected the role of epigenetic modifications in γ-globin expression, harnessing the programmability and versatility of different dCas9-based epigenome editors to evaluate the effect of DNA methylation, histone acetylation or the combination of both epigenetic modifications on the expression of the *HBG* genes. The dCas9-Tet1 editor, previously reported to efficiently mediate DNA demethylation at promoter regions in various cell types [55, 71, 72], was exploited to target DNA demethylation specifically at the *HBG* promoter regions in HSPC-derived erythroid cells. This approach revealed a direct cause-and-effect relationship between promoter methylation and γ-globin gene repression. One can hypothesize that CpG demethylation favors *HBG* expression by hindering the recruitment of the NuRD repressor complex at the *HBG* promoters via MBD2.

It is also plausible that the large dCas9 fusion proteins could transiently displace transcriptional repressors such as LRF or BCL11A due to steric hindrance; however, while such displacement may contribute to HbF reactivation at early time points, it likely does not play a major role at later stages (e.g. day 13 of erythroid differentiation) when neither dCas9 nor its fusion proteins are still present.

A previous study suggested that DNA methylation at the *HBG* promoters does not play a primary role in HbF silencing, as depletion of BCL11A resulted in HbF reactivation without a concomitant decrease in promoter methylation [23]. In contrast, our findings support a model in which HbF silencing is mediated by multiple mechanisms, potentially involving the NuRD complex. These include not only the recruitment of transcriptional repressors such as BCL11A, but also epigenetic modifications, such as DNA methylation.

Furthermore, earlier studies demonstrated γ-globin reactivation in cell lines using epigenome editors that introduced H3K27ac marks (such as p300 and CBP) [54, 56–58]. In line with these findings, our experiments using dCas9-CBP showed significant γ-globin reactivation not only in cell lines but also in erythroid cells derived from epigenome-edited HSPCs, likely by generating an open chromatin state that facilitates the recruitment of factors activating gene expression.

Interestingly, we observed HbF reactivation even though the main repressors, LRF and BCL11A, are still present in these cells. LRF and BCL11A might no longer bind to the *HBG* promoters due to competition (via steric hindrance) with activating factors such as GATA1 at the −189 site and NF-Y at the −115 site [73], once the epigenetic barriers to chromatin occupancy are removed and an open chromatin state is established. Alternatively, LRF and BCL11A may still bind the *HBG* promoters but fail to stably recruit the NuRD repressor complex via MBD2 in the absence of CpG methylation.

Collectively, these results demonstrate that γ-globin reactivation can be robustly achieved by modulating either DNA methylation or histone acetylation at the *HBG* promoters. These findings support the hypothesis that gene activation—whether induced by HPFH mutations, recruitment of CBP or transcriptional activators [74], or enforced chromatin looping [75]—is consistently accompanied by DNA demethylation at the *HBG* promoters. This demethylation may occur through similar or distinct molecular mechanisms, which remain to be fully elucidated.

Of note, at early time points, delivery of the CBP + Tet1 combination resulted in a two-fold increase in γ-globin reactivation compared to Tet1 delivery alone. This observation suggests that while CBP can directly activate gene expression in early hematopoietic progenitors, Tet1 might need the presence of factors (e.g. transcription factors) expressed later in the erythroid differentiation to reactivate HbF in a more physiological manner (i.e. in committed erythroid precursors).

Interestingly, analyses of γ-globin reactivation in *in vitro* generated erythroid cells revealed no dramatic increase in γ-globin levels when combining the effects of DNA methylation and histone acetylation compared to the individual treatments. However, samples treated with Tet1 (alone or in combination with CBP) tend to express greater levels of γ-globin, suggesting that DNA demethylation can promote higher and more durable gene activation. Therefore, these conditions were chosen for *in vivo* long-term studies, while we omitted to assay HSPCs treated with CBP alone. Additionally, epigenome editing studies aimed at repressing gene expression showed that the simultaneous modulation of DNA methylation and histone modification can establish epigenetic memory, enabling persistent genetic tuning [52, 53]. Here, we showed that Tet1 (alone or in combination with CBP) can induce a durable gene activation *in vitro* for ∼3 weeks in HSPC-derived erythroid cells after a transient exposure of HSPCs to epigenome editors. Long-term xenotransplantation experiments showed that the epigenome editing procedure does not affect HSC engraftment and multilineage differentiation, with Tet1- or Tet1/CBP-mediated HbF reactivation occurring in a fraction of erythroid cells derived from repopulating HSCs. Thus, this work has shown residual gene activation in a fraction of human HSC-derived hematopoietic cells 16 weeks after transient exposure to the epigenome editors.

Nevertheless, the extent of HbF reactivation observed across numerous cell divisions during *in vivo* HSC commitment and differentiation was lower compared to that observed *in vitro*. Future studies incorporating additional epigenetic modulators are warranted to better elucidate the key epigenetic modifications required to sustain the maintenance of activating epigenetic marks *in vivo* in a larger fraction of cells. It is plausible that the introduction of only two epigenetic modifications may be insufficient to establish mechanisms of epigenetic memory, as has also been observed in the context of repressive strategies where the use of the KRAB domain combined with DNMT3A/L leads to the introduction of multiple epigenetic modifications both at DNA and histone level (e.g. DNA methylation, H3K27, and H3K9 methylation) [52, 53].

Alternatively, epigenome editors might have induced some toxicity *in vivo*, which could affect cell viability and self-renewal capacity of edited HSCs. The RNA-seq analysis showed a modest impact of epigenome editors on HSPCs. In fact, we observed only ∼100 mildly dysregulated genes (average log_2_FC of ∼2) in treated versus control cells. Most of them are involved in RNA sensing pathways, suggesting that the gene dysregulation is mainly due to the delivery of high quantity of RNA in HSPCs rather than broad epigenetic effects at off-target regions. In addition, genome-wide DNA methylation analysis showed no major changes occurring between control and edited cells in regions encompassing the DEGs or the *in silico* predicted off-targets, confirming the specificity of our epigenetic strategy. Future studies will aim to minimize the amount of RNA delivered to cells. By way of example, prior research has shown that DNA methylation can spread across the target region; thus, only 1 or 2 sgRNAs might be sufficient to achieve relevant HbF levels [55, 76]. Furthermore, we could reduce the amount of editor-encoding mRNA or sgRNA and evaluate whether the levels of DNA demethylation are comparable to those achieved with the current strategy, reaching a compromise between effective HbF reactivation and minimal RNA-induced response. As an alternative, ribonucleoprotein delivery could be investigated to mitigate the potential deleterious effects associated with the innate immune response to RNA.

Importantly, our study paves the way for the development of a targeted, efficacious, and safe DSB-free approach for the treatment of β-hemoglobinopathies. Our study highlighted the pivotal role of epigenetic modifications at a relevant therapeutic target and provided evidence of the efficacy of epigenome editors in reactivating the γ-globin genes. However, the complexity of natural epigenetic regulation needs to be further dissected by evaluating the role of alternative histone modifications that can be modulated to ensure stable epigenetic modification of the target site. Alternatively, the delivery of optimized editing components (e.g. containing less immunogenic DNA targeting domains) using liposomes or lipid nanoparticles [77] could allow repeated injections and ensure the persistence of the desired epigenetic modifications *in vivo*.

In conclusion, this work provides basic knowledge on epigenetic regulation of the γ-globin genes and paves the way for the development of epigenome editing strategies to treat β-hemoglobinopathies.

## Supplementary Material

gkaf637_Supplemental_Files

## Data Availability

RNA-seq data are available in the Gene Expression Omnibus repository under the accession number GSE283100. The raw long-read sequencing data are available at the European Nucleotide Archive (ENA) database (www.ebi.ac.uk/ena/browser) with the accession number PRJEB83576.
