# Supplementary material for: Dissecting the epigenetic regulation of the fetal hemoglobin genes to unravel a novel therapeutic approach for β-hemoglobinopathies
Source: Nucleic Acids Res. 2025 Jul 10;53(13):gkaf637. doi: 10.1093/nar/gkaf637 (PMC12242770; doi:10.1093/nar/gkaf637)
Supplement: gkaf637_Supplemental_Files [file gkaf637_supplemental_files.zip › AmistadiFontana_supplementaryfigures.pdf]

Supplementary Figure S1

A

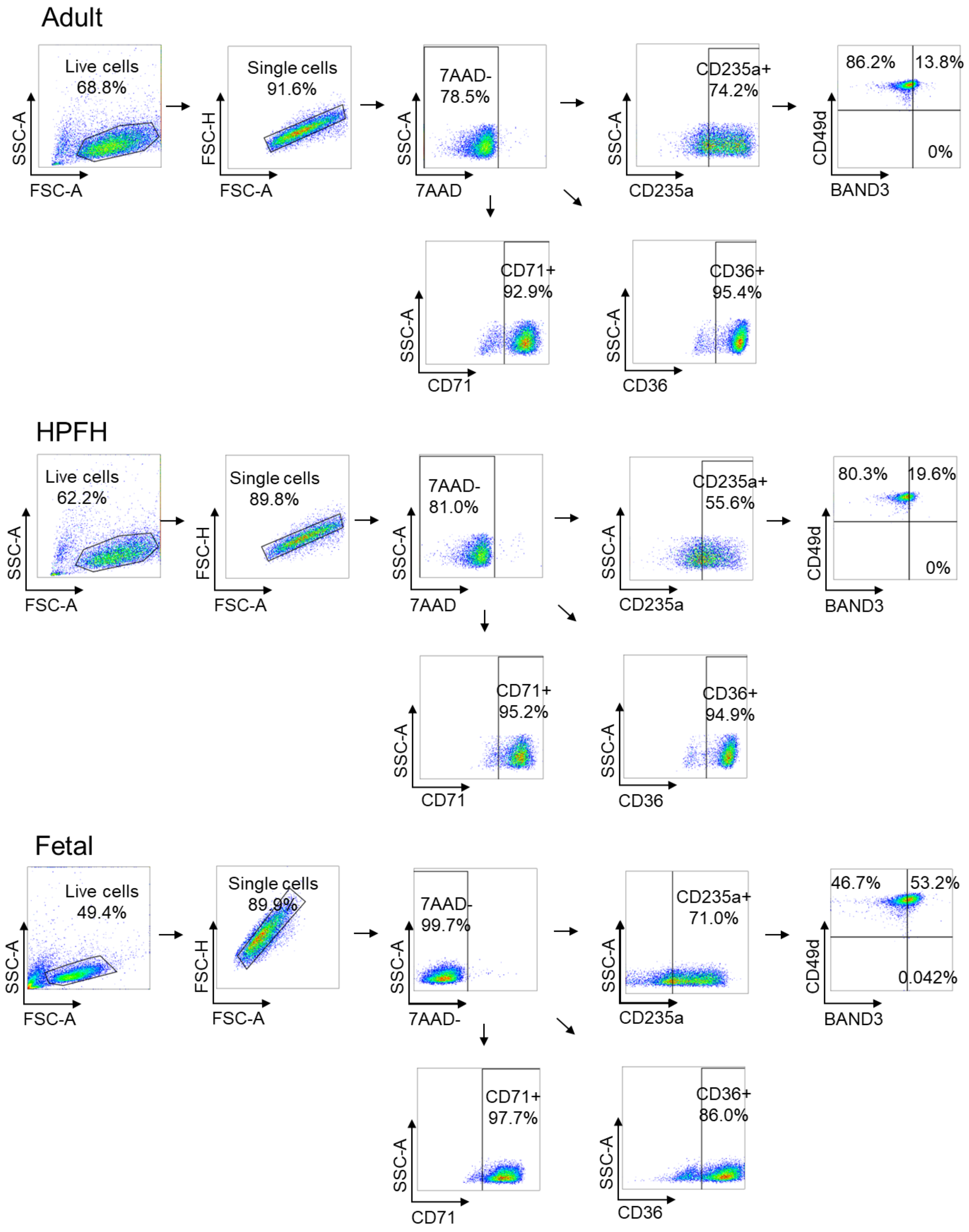

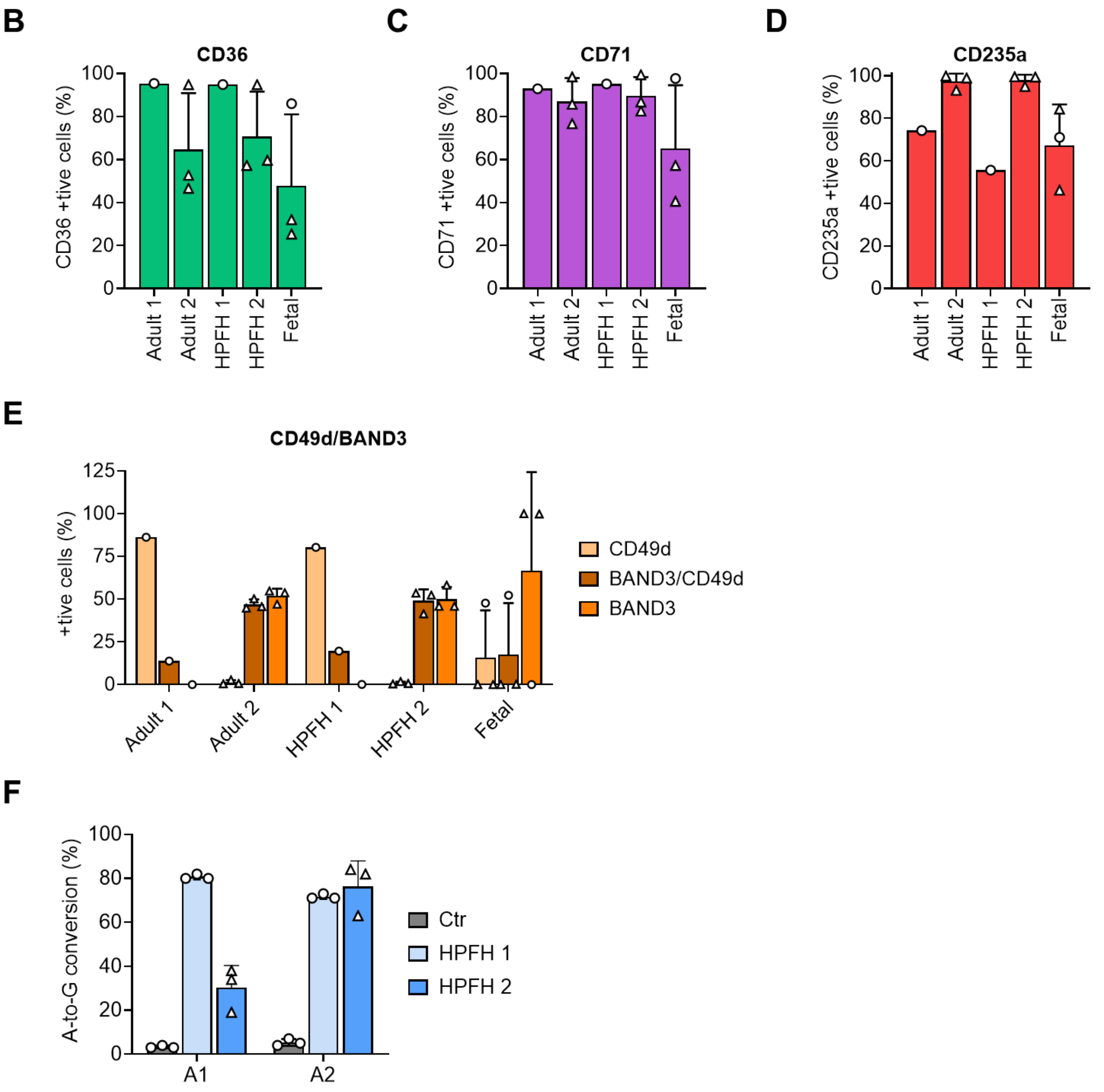

Supplementary Figure S1. Flow cytometry characterization of erythroid cells and editing efficiency in HPFH cells. (**A**) Representative gating strategy used to characterize erythroid cells. We gated on single, live (7AAD-) cells to determine CD235a, CD36, CD71, BAND3 and α4-Integrin expression via flow cytometry. The top panel represents adult cells (Adult); the middle panel represents adult cells edited to recreate HPFH mutations (HPFH); the low panel represents fetal cells (Fetal). This gating strategy was used to analyze data shown in Supplementary Figure S1B-E, S3A-C, S3E. (**B-D**) Frequency of (**B**) CD36+, (**C**) CD71+ and (**D**) CD235a+ cells measured in adult erythroblasts at day 6 of the erythroid differentiation (Adult 1 and HPFH1), adult erythroblasts at day 13 of the erythroid differentiation (Adult 2 and HPHF2), and fetal erythroblasts (Fetal) at day 6 (circle) or day 13 (triangle) of erythroid differentiation. (**E**) Frequency of CD49d+, CD49d+/BAND3+and BAND3+ cells in 7AAD-/CD235+ cells measured by flow cytometry. (**F**) A-T to G-C base-editing efficiency of A1 and A2 of the -200 region of the *HBG* promoters in HPFH samples (see Figure 1C), calculated by the EditR software in samples subjected to Sanger sequencing at day 6 (circle) and 13 (triangle) of erythroid differentiation. Ctr, adult cells transfected with TE buffer only. Data are expressed as mean ± SD (n = 3 biologically independent experiments, 3 donors).

Supplementary Figure S2

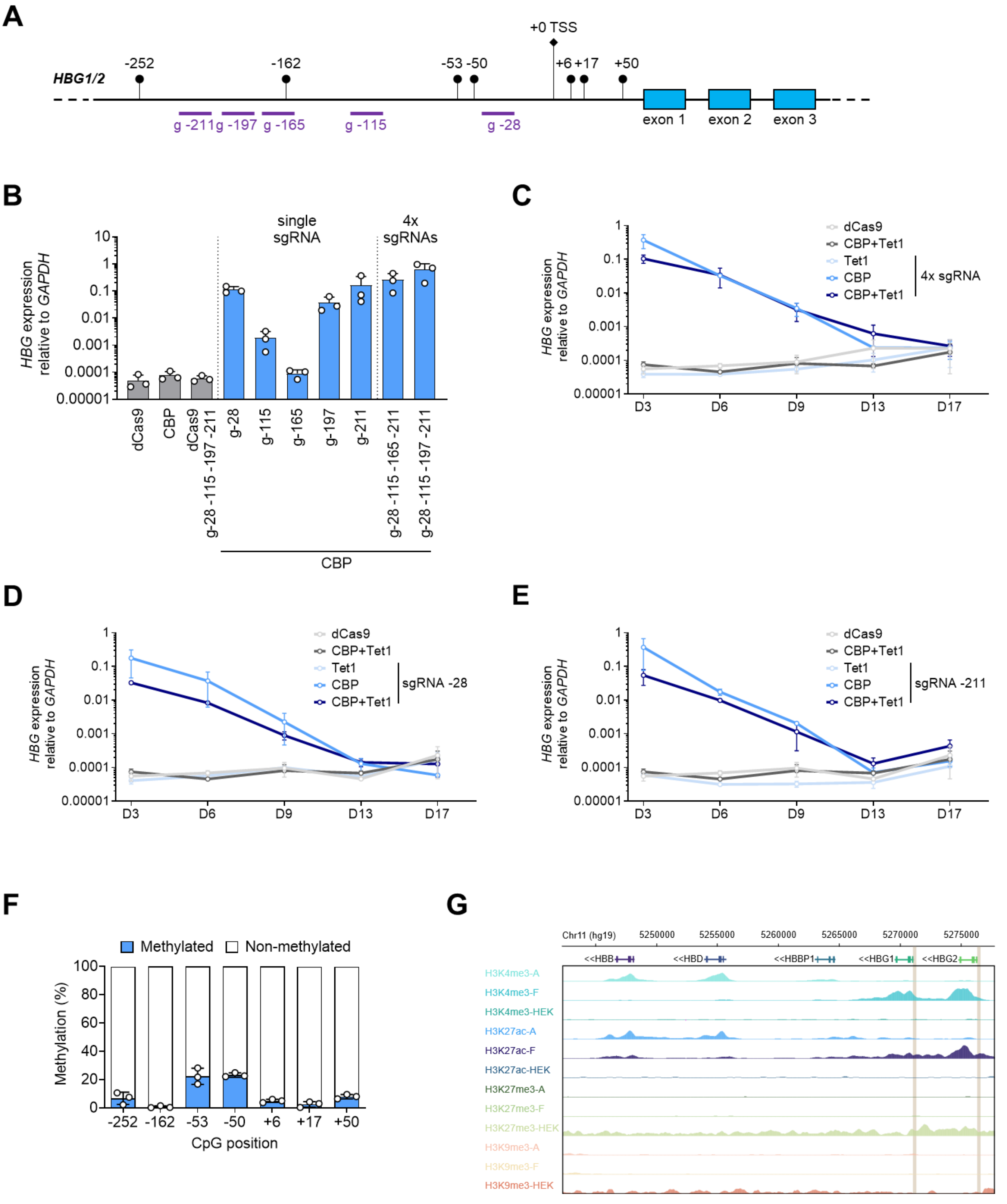

Supplementary Figure S2. Epigenetic reactivation of *HBG* in the HEK293T cell line. (**A**) Schematic representation of the *HBG1/2* gene. Blue boxes depict exons. Purple lines represent sgRNAs (named after the distance in nucleotides of the cleavage site from the TSS). Pins represent the CpGs. (**B**) Relative expression of *HBG* genes normalized to *GAPDH* expression measured by RT-qPCR. Cells were transfected with plasmids expressing the editors alone or together with plasmids expressing the different sgRNAs and analyzed 3 days after transfection. Data are expressed as means ± SD from 3 independent experiments. (**C-E**) Time course analysis of *HBG* expression at day 3, 6, 9, 13, 17 after transfection of the editors and 4xsgRNAs, sgRNA -28, sgRNA-211. Data are expressed as means ± SEM from 2 independent experiments. (**F**) Methylation analysis of CpGs within the *HBG* promoters by bisulfite sequencing in untreated HEK293T. Data are expressed as means ± SD from 3 independent experiments. (**G**) ChIP-seq analysis of *HBG*1/2 promoters (highlighted) as reported in Figure 1A with the addition of tracks for HEK293T cells. Epigenetic modifications include H3K27 acetylation (H3K27ac), H3K4 trimethylation (H3K4me3), H3K27 trimethylation (H3K27me3), H3K9 trimethylation (H3K9me3) in adult (A), fetal erythroblasts (F) and HEK293T (HEK) cells.

Supplementary Figure S3

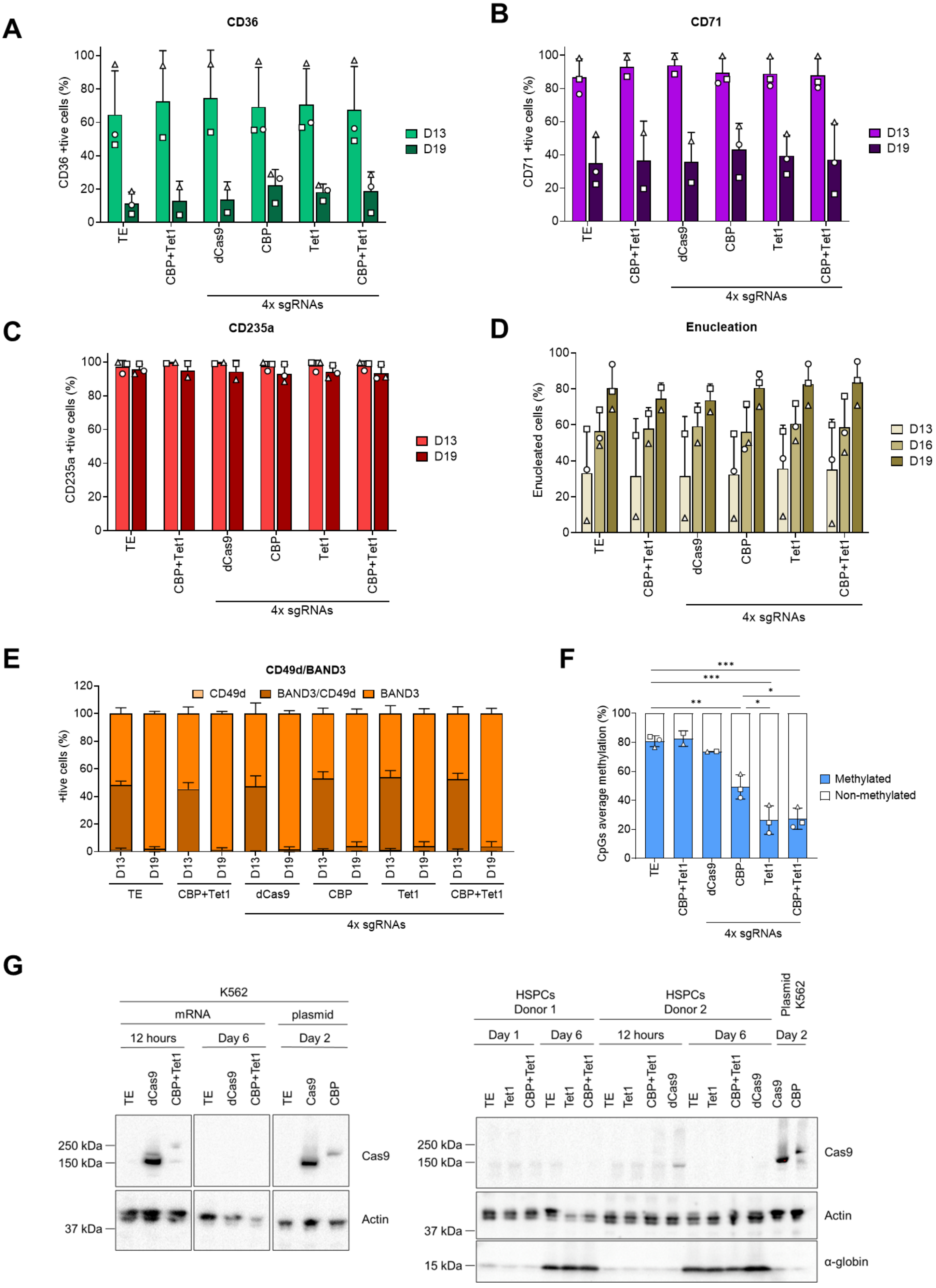

H

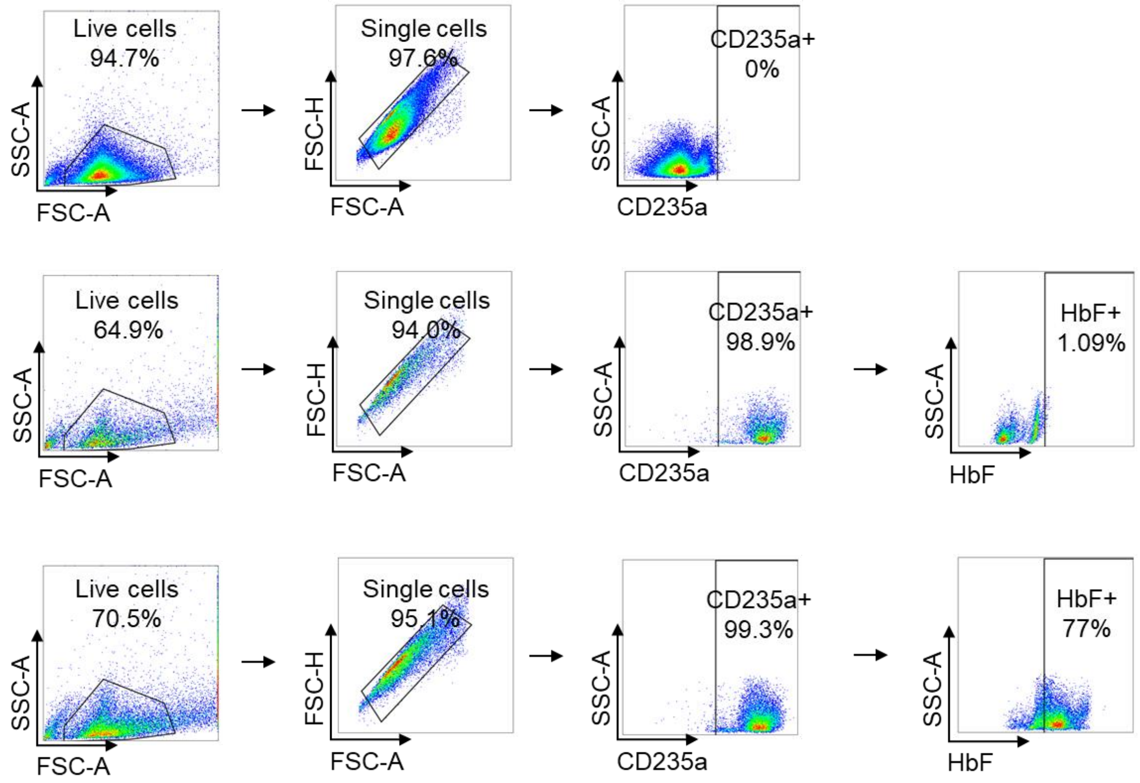
Live cells
94.7%
SSC-A
FSC-A
Single cells
97.6%
FSC-H
FSC-A
CD235a+
0%
SSC-A
CD235a
Live cells
64.9%
SSC-A
FSC-A
Single cells
94.0%
FSC-H
FSC-A
CD235a+
98.9%
SSC-A
CD235a
HbF+
1.09%
SSC-A
HbF
Live cells
70.5%
SSC-A
FSC-A
Single cells
95.1%
FSC-H
FSC-A
CD235a+
99.3%
SSC-A
CD235a
HbF+
77%
SSC-A
HbF

I

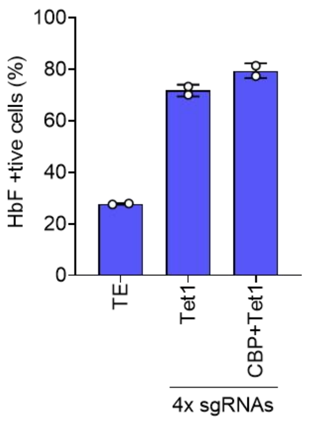
HbF +tive cells (%)
100
80
60
40
20
0
TE
Tet1
CBP+Tet1
4x sgRNAs

J

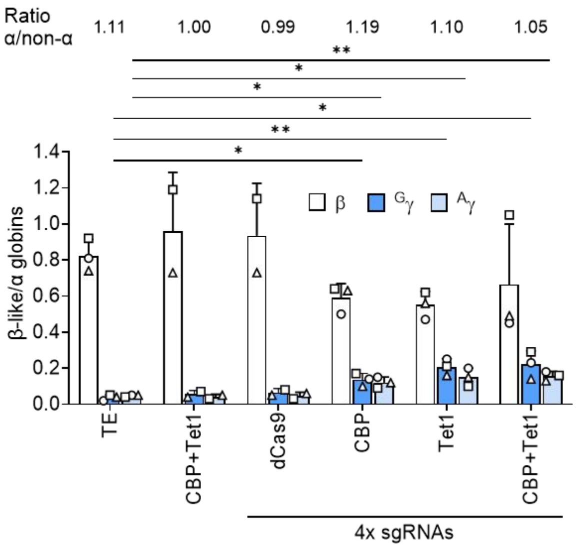
Ratio
α/non-α
1.11
1.00
0.99
1.19
1.10
1.05
**
*
*
*
**
*
β-like/α globins
1.4
1.2
1.0
0.8
0.6
0.4
0.2
0.0
β
Gγ
Aγ
TE
CBP+Tet1
dCas9
CBP
Tet1
CBP+Tet1
4x sgRNAs

Supplementary Figure S3. Erythroid differentiation of HSPCs is not impacted by epigenome editing. Frequency of (**A**) CD36+, (**B**) CD71+ and (**C**) CD235a+ cells at day 13 and 19 of erythroid differentiation, as measured by flow cytometry analysis in control and edited samples. Control cells (TE) were electroporated with TE buffer. (**D**) Frequency of enucleated cells at day 13, 16, and 19 of erythroid differentiation as measured by flow cytometry analysis of DRAQ5 nuclear staining. (**E**) Frequency of CD49d+, BAND3+ and CD49d+/BAND3+ in 7AAD-/CD235+ cells at day 13 and 19 of erythroid differentiation as measured by flow cytometry. (**F**) Average methylation of CpGs withing the HBG promoters by bisulfite sequencing at day 13 of erythroid differentiation. All data are expressed as means ± SD from 3 independent experiments. Each symbol represents a different donor. (**G**) Western blot analysis of Cas9 expression in the K562 cell line (left panel) or in HSPCs from 2 healthy individuals (right panel). Cells treated with epigenome editors were collected at an early time point (12 or 24 hours) and at a later time point (Day 6 after electroporation). As positive control, we electroporated K562 cells with plasmids expressing Cas9 or CBP-dCas9 enzymes and collect them after 48 hours. Actin was used as a loading control. α-globin was used in HSPCs and HSPC-derived erythroblasts as a differentiation control. At the early time points dCas9 and the dCas9 fusions were detected in K562, but not in HSPCs. (**H**) Representative gating strategy for population analysis on live, single erythroid cells at day 19 of the differentiation in unstained (top panel), CD235a-mono-stained (middle panel) and stained cells (low panel) in order to determine HbF expression via flow cytometry. This gating strategy was used to analyze data shown in Figures 2F-G, 5G, S6B, S7B. Within the HbF-negative population, two distinct cell populations are observed, likely due to differences in autofluorescence between enucleated and nucleated cells. (**I**) Percentage of HbF-positive cells measured by flow cytometry at day 6 of erythroid differentiation from cells treated as described in panel G (2 HD donors). (**J**) Expression of β-, Gγ-, and Aγ-globin chains measured by RP-HPLC in RBCs. β-like-globin expression was normalized to α-globin. The α-/non–α-globin ratio is reported on top of the graph. All data are expressed as means ± SD from 3 independent experiments. Each symbol represents a different donor. Asterisks indicate level of statistical significance; *P≤0.05; **P≤0.01; ***P≤0.001; no asterisk = not significant (Unpaired t-test).

Supplementary Figure S4

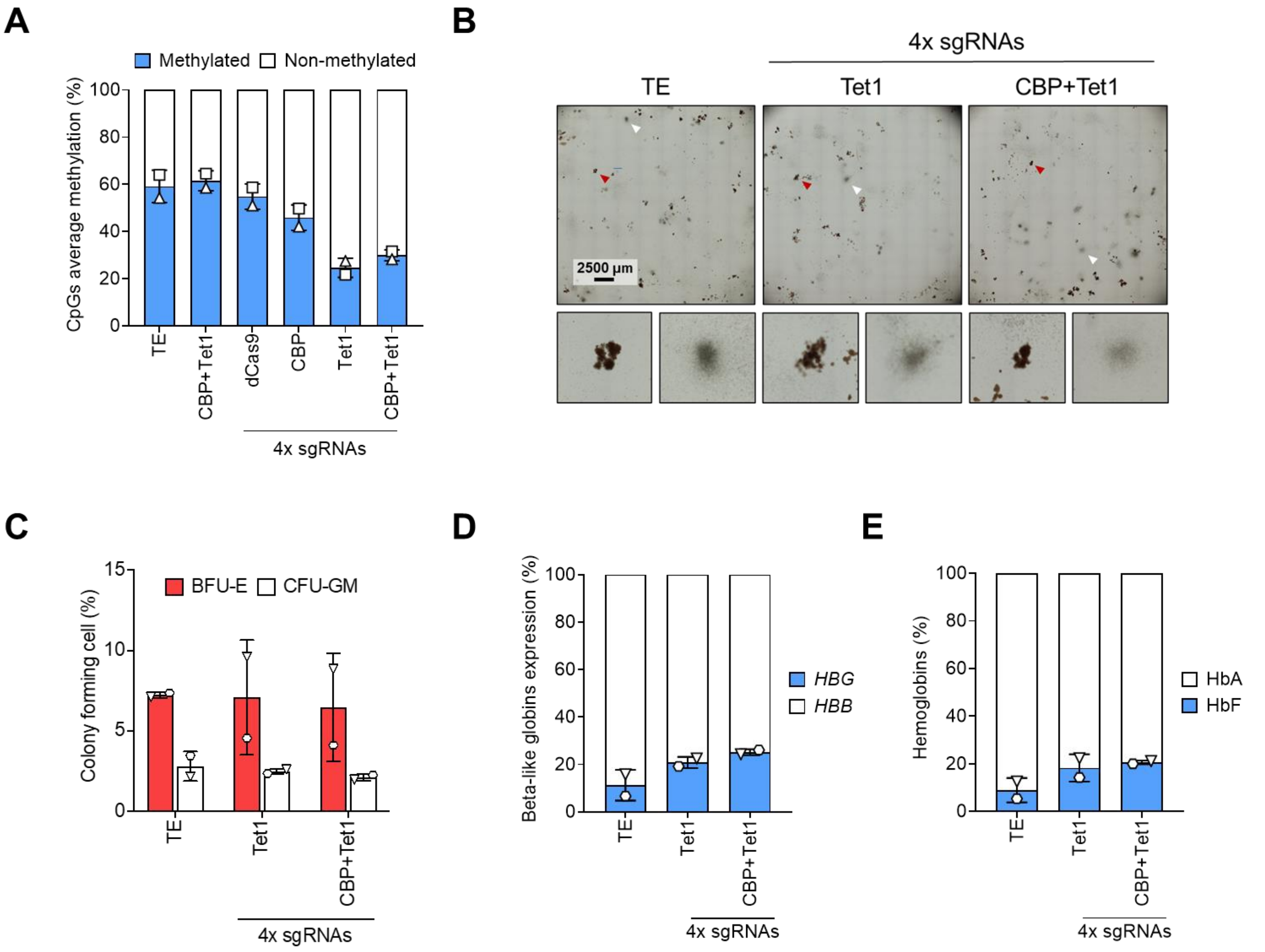

Supplementary Figure S4. Progenitor counts and γ-globin reactivation in BFU-Es after epigenome editing. (**A**) Average methylation of CpGs withing the *HBG* promoters by bisulfite sequencing of a pool of bulk BFU-E. Data are expressed as means ± SD from 2 independent experiments. Each symbol represents a different donor. (**B**) Representative picture of CFC assay after 14 days of culture in a semi-solid media for control cells (TE) and epigenome edited cells. Lower panel with magnification of the two principal types of colonies observed (red arrow BFU-E, left and white arrow CFU-GM, right). (**C**) Colony forming cell frequency in control and edited samples. (**D**) Percentage of β-like globin mRNA (*HBG* and *HBB*) expression measured by RT-qPCR in pools of bulk BFU-E. β-like globin expression was normalized to *HBA*. (**E**) HbF and HbS levels measured by CE-HPLC in pool of bulk BFU-E. The percentage of each Hb type was calculated over the total Hb tetramers. Data are expressed as means ± SD from 2 independent experiments.

Supplementary Figure S5

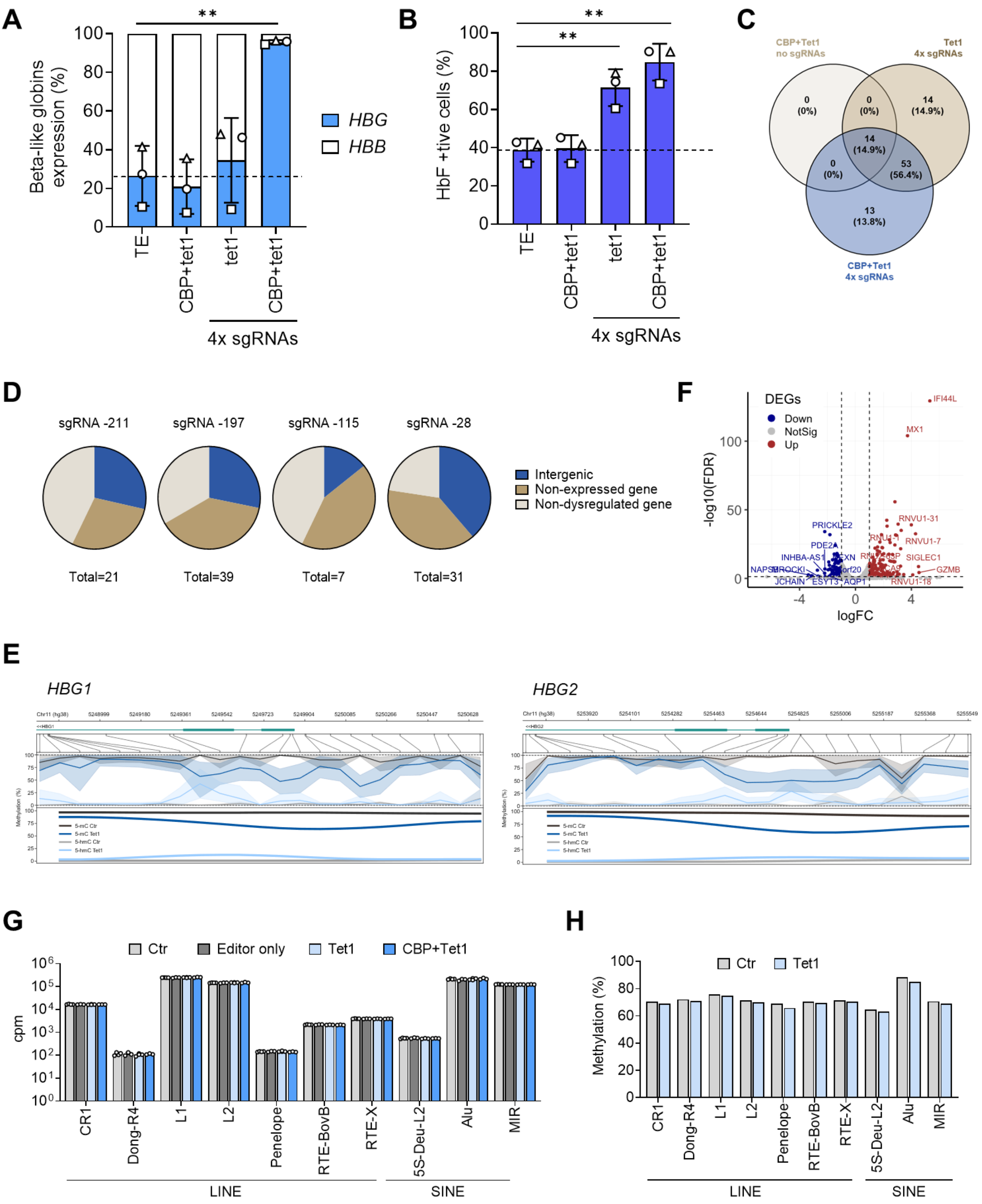

Supplementary Figure S5. Transcriptome and DNA methylome profiles of epigenome-edited HSPCs. (**A**) Percentage of β-like globins (*HBG* and *HBB*) expression normalized to *HBA* expression measured by RT-qPCR. Analysis performed 3 days after electroporation (3 donors SCD HSPCs). (**B**) Percentage of HbF-positive cells measured by flow cytometry at day 6 of erythroid differentiation (3 donors SCD HSPCs). (**C**) Venn diagram reporting the distribution of upregulated genes between the different conditions. (**D**) *In silico* predicted off-target sites for each of the sgRNA. Off-targets were classified as intergenic or intragenic. The list of off-target genes has been cross-referenced with the list of DEGs. The predicted off-target genes were either not expressed or not dysregulated. (**E**) CpG methylation profiles in a magnified view of a ±1-kb region centered on the *HBG2* (above) and *HBG1* (below) promoter region. Individual dots indicate the average methylation of each CpG. Linear regression was applied to the smoothed curve. (**F**) Volcano plots from RNA-seq analyses showing differential gene expression between controls and cells treated with Tet1-4xsgRNAs. RNA-seq was performed 72 h after electroporation in a healthy donor. The horizontal dashed line indicates the threshold on the false discovery rate (FDR ≤ 0.05), and the vertical dashed lines correspond to the threshold on log2FC ≥ 1 or ≤ -1. Upregulated genes are indicated in red and downregulated genes in blue. Genes in grey are not differentially expressed according to the applied thresholds. We observed 163 upregulated and 98 downregulated genes (log2FC≥ 1 or ≤ -1; FDR ≤ 0.05). (**G**) Expression levels of LINE and SINE elements, in control and edited conditions. Expression values are reported as counts per million (cpm). (**H**) DNA methylation levels (%) in LINE and SINE in control and Tet1-edited conditions.

Supplementary Figure S6

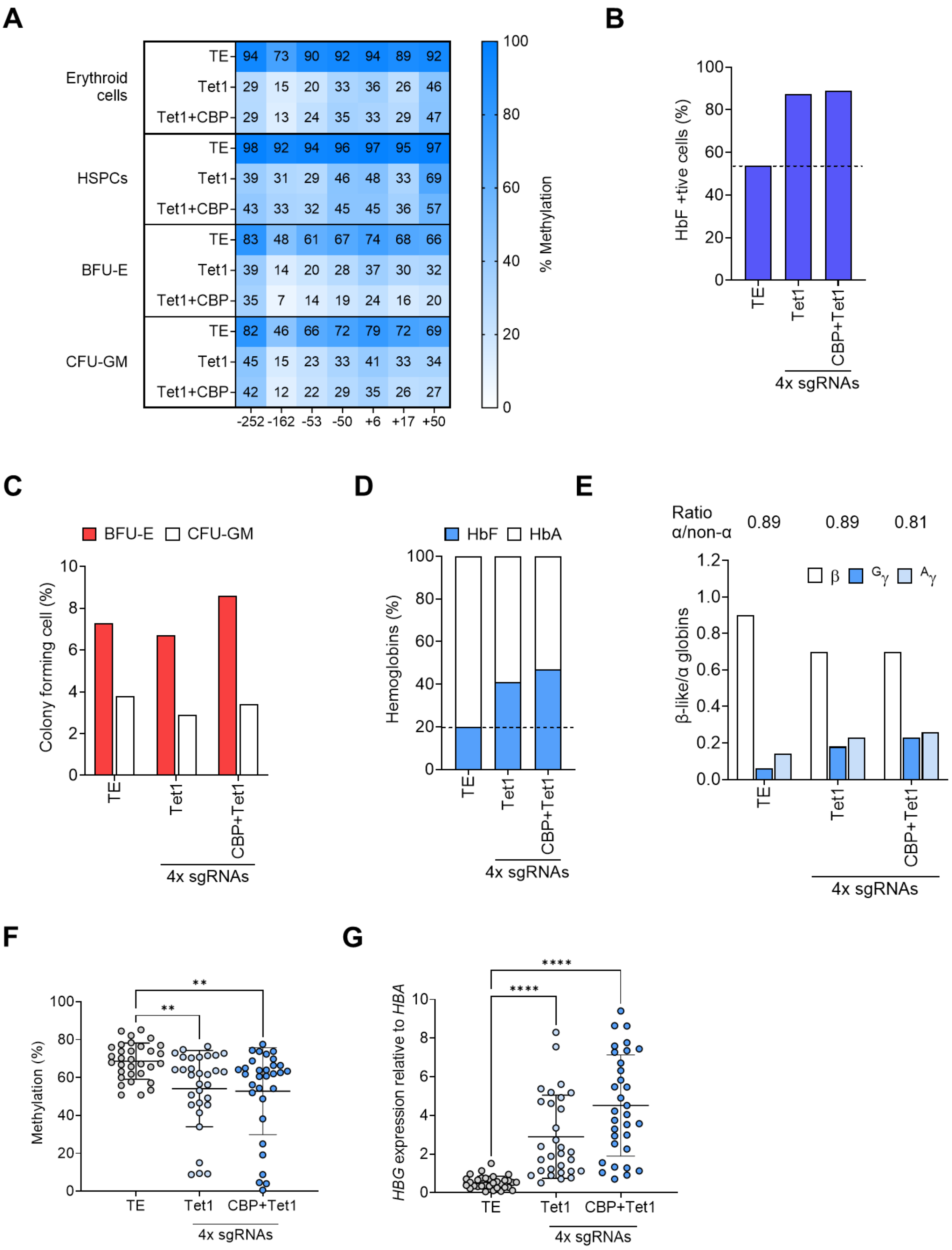

Supplementary Figure S6. Edited HSPCs prior to engraftment in NBSGW immunodeficient mice. (**A**) Methylation analysis of CpGs within the *HBG* promoters by bisulfite sequencing in HSPCs and early erythroid cells 6 days after electroporation. (**B**) Percentage of HbF-positive cells measured by flow cytometry in early erythroid populations. (**C**) Colony-forming cell frequency in control and edited samples 14 days after electroporation. (**D**) HbF and HbS levels measured by CE-HPLC in pools of BFU-E colonies (>25 colonies). The percentage of each Hb type was calculated over the total Hb tetramers. (**E**) Expression of β-, $^{G}\gamma$-, and $^{A}\gamma$-globin chains measured by RP-HPLC in pools of BFU-E colonies. β-like-globin expression was normalized to α-globin. The α-/non–α-globin ratio is reported on top of the graph. (**F**) Average methylation of CpGs withing the *HBG* promoters by bisulfite sequencing in single BFU-E colonies differentiated from control and epigenome edited HSPCs. Data are expressed as means ± SD. (**G**) Percentage of β-like globin mRNA (*HBG* and *HBB*) expression measured by RT-qPCR in single BFU-E colonies differentiated from control and epigenome edited HSPCs. β-like globin expression was normalized to *HBA*. Data are expressed as means ± SD. Asterisks indicate level of statistical significance; **P≤0.01; ****P≤0.0001; no asterisk = not significant (Unpaired t-test).
