# Supplementary material for: Dissecting the epigenetic regulation of the fetal hemoglobin genes to unravel a novel therapeutic approach for β-hemoglobinopathies
Source: Nucleic Acids Res. 2025 Jul 10;53(13):gkaf637. doi: 10.1093/nar/gkaf637 (PMC12242770; doi:10.1093/nar/gkaf637)
Supplement: gkaf637_Supplemental_Files [file gkaf637_supplemental_files.zip › Supplementary Table legends.docx]

**Supplementary Table S1.** Differentially expressed genes in SCD HSPCs electroporated with CBP+Tet1, Tet1-4xsgRNA, CBP+Tet1-4xsgRNA vs mock-electroporated SCD HSPCs.

**Supplementary Table S2.** *In silico* predicted sgRNA-dependent off-target sites (sgRNA -211, -197, -115, -28).

**Supplementary Table S3.** Differentially expressed genes in HD HSPCs electroporated with Tet1-4xsgRNAs vs mock-electroporated HD HSPCs.

**Supplementary Table S4.** DNA methylation levels in upregulated and down-regulated genes in control and treated samples.

**Supplementary Table S5.** DNA methylation levels in all genes in control and treated samples.

**Supplementary Table S6.** DNA methylation levels of *in silico* predicted sgRNA-dependent off-target sites in control and treated samples.

**Supplementary Table S7.** Expression levels (counts per million mapped reads) in repeat elements in control and treated samples and DNA methylation levels in repeat elements in control and treated samples.
